# Cardiomyocyte-derived YOD1 promotes pathological cardiac hypertrophy by deubiquitinating and stabilizing STAT3

**DOI:** 10.1126/sciadv.adu8422

**Published:** 2025-06-25

**Authors:** Bozhi Ye, Wante Lin, Yucheng Jiang, Zhaozheng Zheng, Yanhong Jin, Diyun Xu, Yingjie Liao, Zhihan Jia, Jiaji Chen, Gaojun Wu, Peiren Shan, Guang Liang

**Affiliations:** ^1^ Department of Cardiology and the Key Laboratory of Cardiovascular Disease of Wenzhou, the First Affiliated Hospital, Wenzhou Medical University, Wenzhou, Zhejiang, China.; ^2^ School of Pharmaceutical Sciences, Hangzhou Medical College, Hangzhou, Zhejiang, China.; ^3^ Chemical Biology Research Center, School of Pharmaceutical Sciences, Wenzhou Medical University, Wenzhou, Zhejiang, China.

## Abstract

Identifying previously unknown targets for pathological cardiac hypertrophy and understanding its mechanisms are crucial. Here, we observed that the deubiquitinating enzyme YOD1 was moderately elevated in human hypertrophic myocardium and mouse models. Cardiomyocyte-specific knockout of YOD1 reduced Ang II– and TAC-induced cardiac hypertrophy. Subsequently, we used multiple proteomic analyses to identify and confirm STAT3 as a substrate protein for YOD1. Mechanistically, our findings revealed that the C155 site of YOD1 removes K48-linked ubiquitin chains from K97 on STAT3, stabilizing STAT3 levels and enhancing its nuclear translocation in cardiomyocytes under Ang II stimulation. Notably, inhibiting STAT3 reversed the antihypertrophic effects of YOD1 deficiency in Ang II–challenged mice. In addition, pharmacological inhibition of YOD1 mitigated Ang II–induced pathological ventricular remodeling in mice. This study clarifies the role of YOD1 and introduces a previously unidentified YOD1-STAT3 axis in regulating pathological cardiac hypertrophy, providing valuable insights for drug development targeting this condition.

## Teaser

YOD1 promotes pathological cardiac hypertrophy by deubiquitinating and stabilizing STAT3 protein.

## INTRODUCTION

Pathological cardiac hypertrophy represents a crucial pathological foundation for heart failure and serves as an independent risk factor contributing to the increased morbidity and mortality associated with cardiovascular diseases (*1*, *2*). Although some antihypertrophy agents targeting angiotensin II (Ang II) and β-adrenergic receptors are clinically used, their efficacy in mitigating cardiac hypertrophy remains limited (*3*). Investigating the regulatory molecules and mechanisms involved in pathological cardiac hypertrophy is of notable scientific importance and clinical relevance and may yield potential targets for innovative treatments for managing cardiac hypertrophy effectively.

Ubiquitination/deubiquitination modification serves as an essential mechanism for protein posttranscriptional modification and plays a crucial role in various biological actions. The process of deubiquitination is mediated by deubiquitinating enzymes (DUBs). DUBs reverse the ubiquitination by removing ubiquitin molecules from substrate proteins to maintain the stability of substrates. Cardiomyocytes, as terminally differentiated cells with limited regenerative capacity, require the maintenance of key protein stability to safeguard their functionality (*4*). Recent studies have shown that several DUBs participate in regulating the pathophysiology in cardiovascular diseases (*5*). DUBs are categorized into seven families, among which the Ovarian Tumor (OTU) family is the second-largest DUB family. Members of the OTU family have a highly conserved cysteine protease domain known as the OTU domain (*6*). The OTU family has been implicated in various diseases, including immune system disorders (*7*), neurological diseases (*8*), and cancer (*9*). In recent years, increasing attention has been directed toward the role of the OTU family in cardiac pathology. Research indicates that OTUB1 plays a significant role in regulating doxorubicin-induced cardiomyopathy (*10*) and diabetic cardiomyopathy (*11*). We previously demonstrated that OTUD1 is involved in cardiac hypertrophy induced by Ang II (*12*). DUBs in the OTU family emerge as potential and fascinating targets for the treatment of pathological cardiac hypertrophy.

Ubiquitin (Ub) thioesterase OTU1 (YOD1) is a crucial member of the OTU family and was initially identified by Ernst *et al.* (*13*). Recent studies have indicated that YOD1 is implicated in the onset and progression of various cancer types, including breast cancer (*14*), hematologic malignancies (*9*), and pancreatic cancer (*15*). However, its role in cardiovascular diseases has yet to be documented. Through screening of the Gene Expression Omnibus (GEO) database followed by polymerase chain reaction (PCR) validation, we found that YOD1 expression in cardiomyocytes is up-regulated in pathological cardiac hypertrophy, indicating the involvement of YOD1 in this disease. In this study, we further explored the role and regulating mechanism of YOD1 in cardiac hypertrophy. We revealed that either cardiomyocyte-specific YOD1 knockout (YOD1CKO) or pharmacological inhibition of YOD1 significantly attenuates Ang II–induced cardiac hypertrophy and dysfunction in mice. Mechanistically, we identified signal transducer and activator of transcription 3 (STAT3) as the substrate protein of YOD1 in cardiomyocytes. YOD1 removes K48-linked Ub chains at the K97 site on STAT3 protein, thereby increasing STAT3 stability. This study highlights the involvement of YOD1 in mediating cardiac hypertrophy, providing a promising therapeutic target for this disease.

## RESULTS

### Identification of YOD1 as a contributor to cardiac hypertrophy

**Fig. 1.**
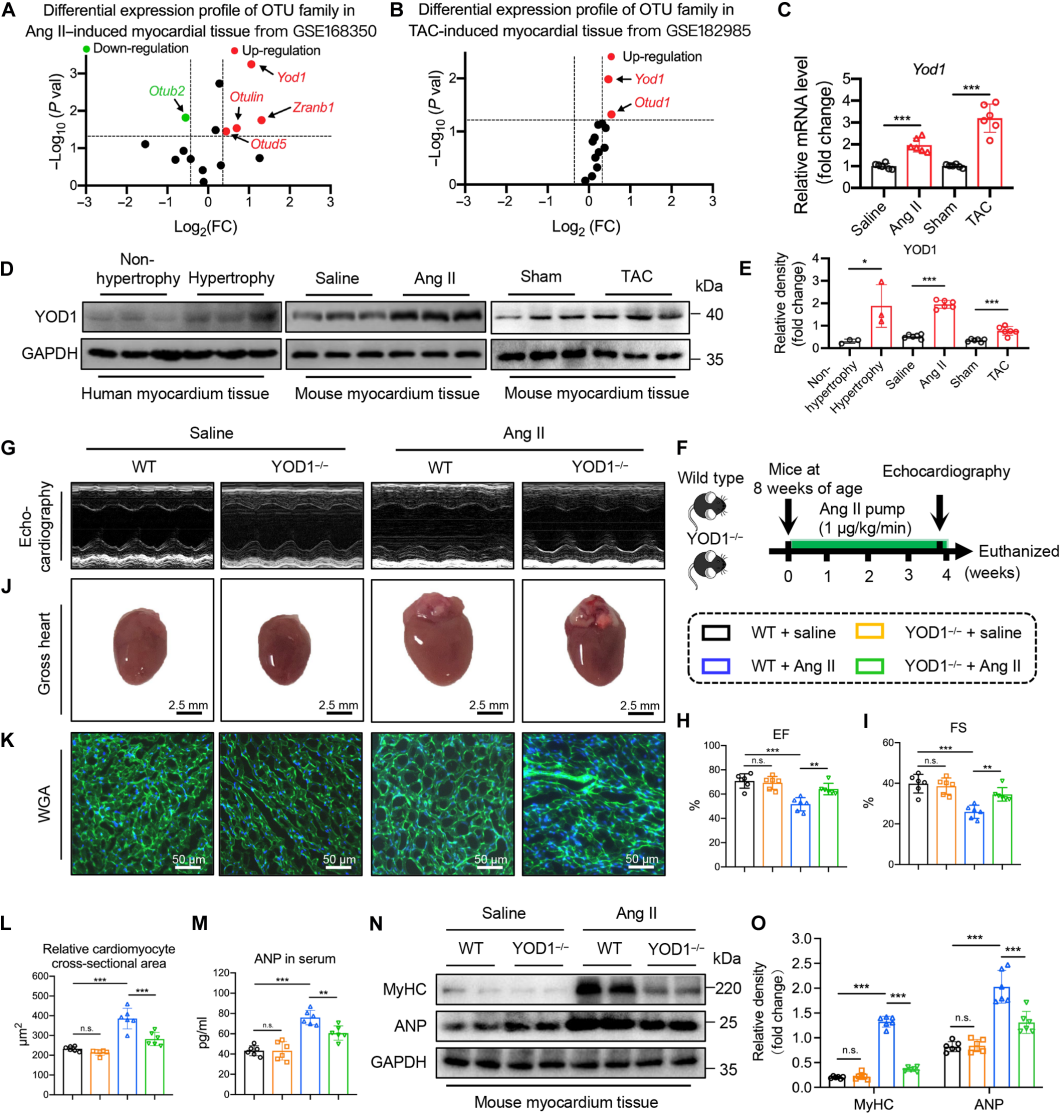
Identification of YOD1 as a contributor to cardiac hypertrophy. (**A** and **B**) RNA transcriptome sequencing was performed to analyze the expression profile of DUBs in the OTU family within mouse myocardium subjected to Ang II– (A) and TAC-induced (B) conditions. The log_2_ fold change (FC) values reflect relative expression changes of OTU family members. Red and green points indicate up-regulated and down-regulated OTUs compared to the control group, while black points represent OTUs without statistically significant differences from the control group. (**C**) Real-time quantitative PCR (qPCR) analysis of mRNA expression levels of OTU family members in mouse myocardium tissue induced by Ang II and TAC stimulation. (**D** and **E**) Representative Western blot analysis of YOD1 in human myocardium tissues from both nonhypertrophic and hypertrophic samples, as well as in mouse myocardium tissues subjected to saline and Ang II stimulation, along with tissues from sham-operated and TAC-treated mice (E) and densitometric quantification (E). (**F**) The experimental flowchart for the Ang II–induced cardiac hypertrophic mouse model. YOD1 whole-body knockout (YOD1^−/−^) mice and wild-type (WT) C57BL/6J mice were injected with Ang II (1 μg kg^–1^ min^–1^) or saline via an osmotic pump for 4 weeks to induce cardiac hypertrophy. (**G**) Representative M-mode echocardiography of mice in each group. (**H** and **I**) Myocardial function parameters, including ejection fraction (EF; H) and fractional shortening (FS; I), were evaluated in mice through echocardiography. (**J**) Representative images of whole hearts. (**K** and **L**) Representative images of wheat germ agglutinin (WGA; K) staining in sections of hearts and quantitative area analysis (L). (**M**) The plasma levels of atrial natriuretic peptide (ANP) in each group. (**N** and **O**) Representative Western blot analysis of MyHC and ANP in myocardium tissues of YOD1^−/−^ and WT mice subjected to saline and Ang II stimulation (N) and densitometric quantification (O). Not significant (n.s.), *P* > 0.05; *P < 0.05; **P < 0.01; ***P < 0.001. *n* = 6 [*n* = 3 for the human myocardium sample in (D)].

We investigated the expression profiles of DUBs in the OTU family from the public GEO database using the hypertrophic myocardial tissues of mice with Ang II infusion (GSE168350; Fig. 1A) and transverse aortic constriction (TAC) surgery (GSE182985; Fig. 1B). Only the *Yod1* mRNA expression level is consistently elevated in these two cardiac hypertrophy models. We then confirmed a substantial increase in *Yod1* mRNA levels in mouse myocardial tissues of these two established models in our lab (Fig. 1C). In addition, the protein levels of YOD1 were markedly increased in myocardial tissues of Ang II– or TAC-challenged mice (Fig. 1, D and E). We also measured YOD1 expression in human hypertrophic myocardium. Patients diagnosed with hypertrophic cardiomyopathy demonstrated significantly elevated protein and mRNA expressions of YOD1 in their myocardium (Fig. 1, D and E, and fig. S1). These data suggest the potential involvement of YOD1 in pathological cardiac hypertrophy.

We initially generated the whole-body YOD1CKO (YOD1^−/−^) mice (fig. S2, A and B). Subsequently, we used Ang II infusion to induce pathological cardiac hypertrophy in both wild-type (WT) and YOD1^−/−^ mice (Fig. 1F). Ang II effectively induced sustained hypertension in mice, and the absence of YOD1 did not alter the blood pressure profile in Ang II–infused mice (fig. S3A). There was no significant difference in body weight between YOD1^−/−^ mice and WT counterparts (fig. S3B). However, YOD1CKO significantly ameliorated cardiac dysfunction induced by Ang II, as evidenced by notable improvements in ejection fraction (EF) and fractional shortening (FS) metrics (Fig. 1, G to I, and table S1). The gross heart morphology, as well as hematoxylin and eosin (H&E) and wheat germ agglutinin (WGA) staining, revealed that YOD1 deficiency mitigated the increase in myocardial cross-sectional area in Ang II–treated mice (Fig. 1, J to L, and fig. S4, A and B). Furthermore, markers indicative of cardiac hypertrophy, including the ratios of heart weight to body weight (HW/BW) and heart weight to tibia length (HW/TL), and the levels of serum atrial natriuretic peptide (ANP) and cardiac Myosin Heavy Chain (MyHC) and ANP proteins were markedly reduced in Ang II–induced YOD1^−/−^ mice compared to those in WT mice (Fig. 1, M to O, and table S1). To assess the impact of YOD1 on myocardial fibrosis, we used Masson’s trichrome and Sirius Red staining, and the results suggested that knocking out YOD1 substantially improved collagen deposition induced by Ang II in mouse hearts (fig. S5, A to D). We also observed significantly decreased collagen type I (Col-I) and transforming growth factor–β (TGF-β) levels in the myocardial tissues of Ang II–induced YOD1^−/−^ mice compared to WT mice (fig. S5, E to G). These findings indicate that YOD1CKO ameliorates Ang II–induced cardiac hypertrophy, fibrosis, and dysfunction. In addition, these data show that YOD1 deficiency does not affect the normal cardiac function in mice under basal conditions.

### Cardiomyocyte YOD1 is highly expressed and promotes cardiomyocyte hypertrophy

**Fig. 2.**
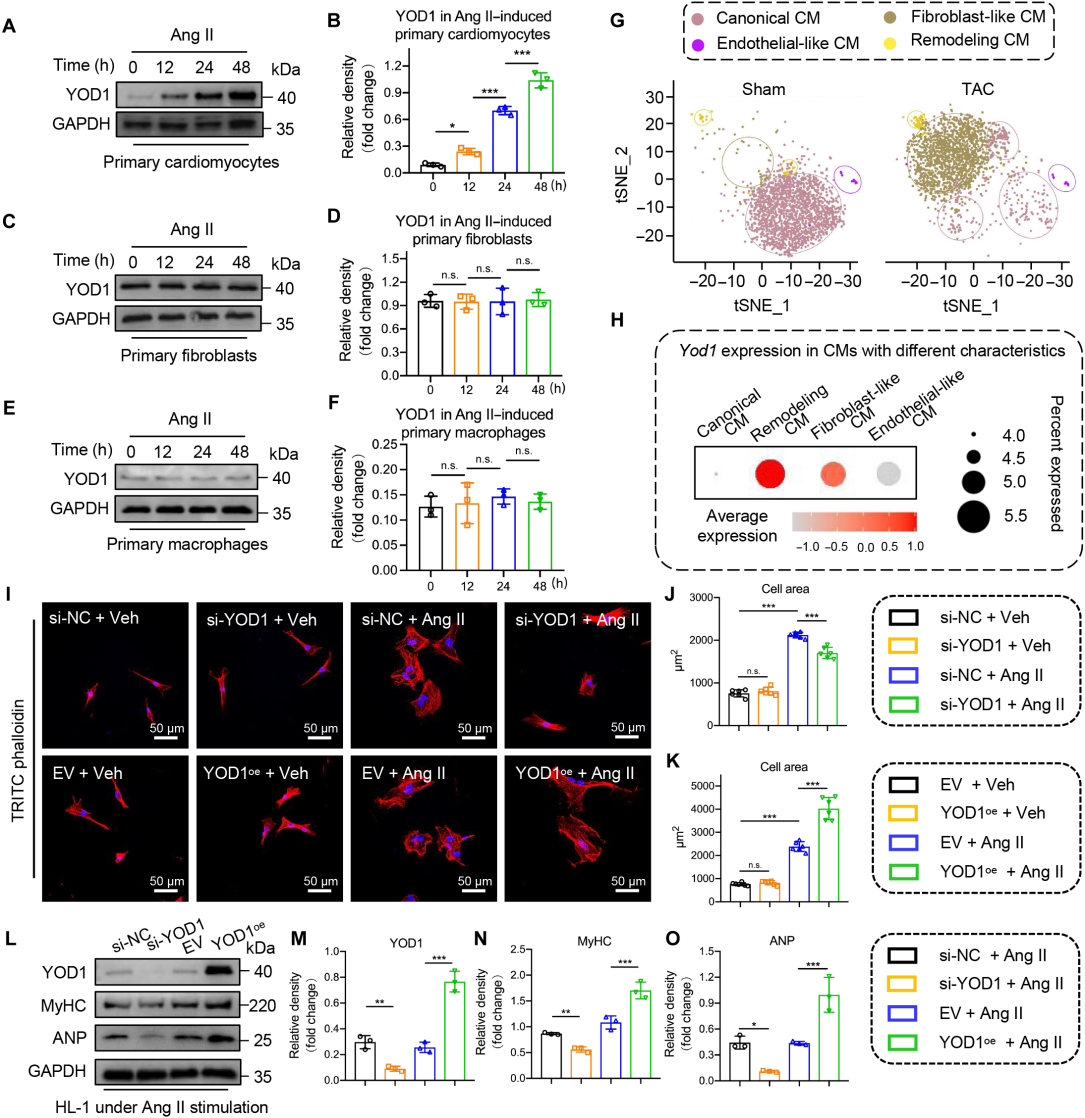
Cardiomyocyte YOD1 is highly expressed and promotes cardiomyocyte hypertrophy. (**A** to **F**) Representative Western blot analysis of YOD1 in primary cardiomyocytes (A), primary fibroblasts (C), and primary macrophages (E) subjected to Ang II stimulation at various time points and densitometric quantification (B, D, and F). *n* = 3. h, hours. (**G**) Single-cell mRNA sequencing was conducted on the hearts of mice subjected to sham operation and TAC treatment. For each group, single-cell suspensions from three to four hearts were pooled into one sample. The t-SNE distribution of clustering identified four distinct functional cardiomyocyte clusters: canonical cardiomyocyte, fibroblast-like cardiomyocyte, endothelial-like cardiomyocyte, and remodeling cardiomyocyte. CM, cardiomyocyte. (**H**) The dot plot illustrates the relative expression of *Yod1* across various functional cardiomyocyte clusters. (**I** to **K**) Representative images of tetramethyl rhodamine isothiocyanate (TRITC)–labeled rhodamine-phalloidin staining in primary cardiomyocytes. Cardiomyocytes were transfected with plasmids containing either the empty vector (EV) or YOD1 overexpression (YOD1^oe^) and siRNAs targeting negative control (si-NC) or YOD1 (si-YOD1), followed by stimulation with Ang II (1 μM for 24 hours) or Vehicle (Veh). The surface area of the cardiomyocytes was assessed using TRITC-labeled rhodamine-phalloidin staining (I), accompanied by a corresponding quantitative analysis (J and K). *n* = 6. (**L** to **O**) Representative Western blot analysis of YOD1, MyHC, and ANP in HL-1 under Ang II stimulation (L) and densitometric quantification (M to O). *n* = 6. The cell processing procedure is consistent with that depicted in (J). *n* = 3. n.s., *P* > 0.05; *P < 0.05; **P < 0.01; ***P < 0.001.

We investigated the cellular distribution of YOD1 in the hypertrophic heart. Initially, a time-dependent increase in the YOD1 protein level was detected in primary cardiomyocytes stimulated by Ang II (Fig. 2, A and B), while this increase was not observed in primary fibroblasts and macrophages (Fig. 2, C to F). Subsequently, we examined the expression of YOD1 in various types of cardiomyocytes through single-cell RNA sequencing (scRNA-seq) of TAC-induced mouse myocardial tissues. scRNA-seq revealed a significant increase in the number of remodeling cardiomyocytes, fibroblast-like cardiomyocytes, and endothelial-like cardiomyocytes in mouse myocardial tissue after TAC surgery (Fig. 2G). Notably, YOD1 expression was markedly higher in remodeling cardiomyocytes compared to other types of cardiomyocytes (Fig. 2H), suggesting that cardiomyocyte YOD1 plays a pivotal role in promoting hypertrophy. To validate our hypothesis, we used small interfering RNA (siRNA)–mediated knockdown of YOD1 in cultured cardiomyocytes and showed that the down-regulation of YOD1 significantly alleviates Ang II–induced hypertrophy, while overexpression of YOD1 in cardiomyocytes via YOD1 plasmid transfection markedly exacerbated Ang II–induced cardiomyocyte hypertrophy (Fig. 2, I to K). In addition, knockdown of YOD1 inhibited the Ang II–induced expression of hypertrophic proteins MyHC and ANP in cardiomyocytes, and overexpression of YOD1 significantly enhanced the levels of MyHC and ANP (Fig. 2, L to O). These findings provide evidence that cardiomyocyte YOD1 plays a crucial role in driving cardiac hypertrophy.

### Cardiomyocyte-specific knockout of YOD1 alleviated the cardiac remodeling

**Fig. 3.**
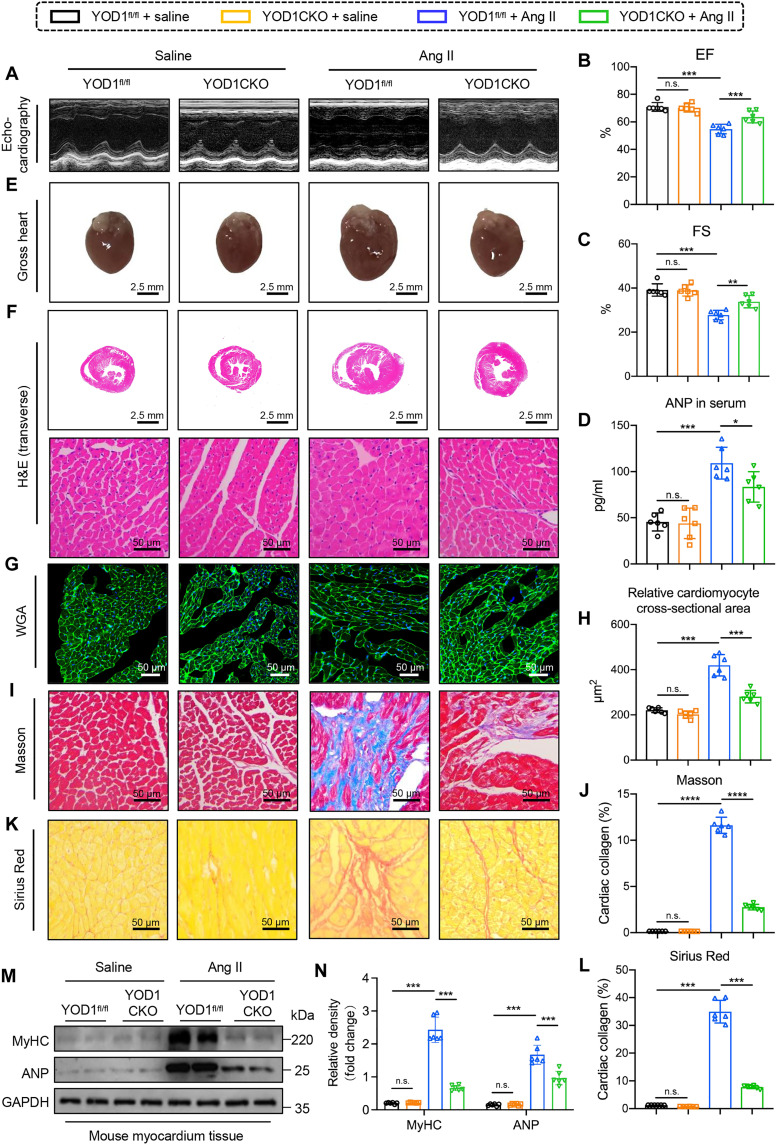
Cardiomyocyte-specific knockout of YOD1 alleviated the cardiac remodeling. Healthy male YOD1CKO mice (cardiomyocyte-specific YOD1CKO mice) aged 6 to 8 weeks and YOD1^fl/fl^ mice were injected with Ang II (1 μg kg^–1^ min^–1^) or normal saline via an osmotic pump (catalog no. ALZET Model 1004, USA) for 4 weeks to induce cardiac hypertrophy. (**A**) Representative M-mode echocardiography of mice in each group. (**B** and **C**) Myocardial function parameters, including EF (B) and FS (C), were evaluated in mice through echocardiography. (**D**) The plasma levels of ANP in each group. (**E**) Representative images of whole hearts. Scale bars, 2.5 mm. (**F**) Representative images of H&E staining of transverse section in myocardium tissues. Scale bars, 2.5 mm and 50 μm. (**G** and **H**) Representative images of WGA (G) staining in sections of hearts and quantitative area analysis (H). Scale bars, 50 μm. (**I** to **L**) Fibrotic areas were evaluated using Masson’s trichrome (I) and Sirius Red (K) staining, as well as the quantification of fibrotic regions (H and J) in heart sections from each group. Scale bars, 50 μm. (**M** and **N**) Representative Western blot analysis of MyHC and ANP (M) and densitometric quantification (N). *n* = 6. n.s., *P* > 0.05; *P < 0.05; **P < 0.01; ***P < 0.001. YOD1^fl/fl^, YOD1^fl/fl^ mice.

We generated cardiomyocyte-specific YOD1CKO mice and confirmed a reduction in YOD1 level in the heart tissue of YOD1CKO mice (fig. S6, A to C). As shown in Fig. 3 (A to C) and table S2, Ang II–induced cardiac dysfunction was significantly reversed in YOD1CKO mice, as evidenced by increased EF and FS. The ratios of HW/BW and HW/TL, as well as serum ANP concentration, were also markedly reduced in YOD1CKO mice compared to those in the YOD1^fl/fl^ counterpart (Fig. 3D and table S2). Histopathological assessments revealed that both the overall size of the heart and the cross-sectional area of cardiomyocytes were decreased by YOD1CKO in Ang II–challenged mice (Fig. 3, E to H, and fig. S7A). Masson’s trichrome staining and Sirius Red staining indicated that YOD1CKO markedly diminished Ang II–induced cardiac fibrosis (Fig. 3, I to L, and fig. S7, B to E). In addition, both mRNA and protein expression levels of MyHC, ANP, Col-I, and TGF-β were significantly reduced in Ang II–infused YOD1CKO mouse hearts (Fig. 3, M and N, and fig. S7, F and G). These data show that cardiomyocyte-specific YOD1 deficiency significantly ameliorates Ang II–induced cardiac remodeling and dysfunction.

We further evaluated the protective effects of YOD1CKO against TAC-induced cardiac hypertrophy. Echocardiographic results indicate that YOD1CKO effectively mitigates the cardiac dysfunction induced by TAC in mice (fig. S8, A to C, and table S3). Examinations on serum ANP level, whole-heart imaging, and H&E and WGA staining revealed that YOD1CKO substantially alleviated TAC-induced cardiac hypertrophy (fig. S8, D to H). Masson’s trichrome and Sirius Red staining demonstrated that mice with YOD1CKO exhibited a significant reduction in ventricular fibrosis compared to WT controls under TAC conditions (fig. S8, I to L). Similar changing trends were observed in the protein levels of MyHC, ANP, Col-I, and TGF-β (fig. S8, M to Q). These findings suggest that YOD1CKO also protects hearts from TAC-induced cardiac remodeling in mice.

We also developed another mouse model of cardiac remodeling, myocardial infarction–induced ventricular hypertrophy, and observed that myocardial-specific YOD1CKO significantly protected hearts in this model. We found that cardiomyocyte-specific YOD1 deficiency improved EF and FS and reduced plasma ANP levels in myocardial infarction–induced mice (fig. S9, A to C, and table S4). Simultaneously, assessments of cardiac gross morphology, H&E staining, WGA staining, and the ratios of HW/BW and HW/TL indicated that myocardial-specific YOD1CKO effectively mitigated hypertrophy induced by myocardial infarction (fig. S9, D to H). Masson’s trichrome and Sirius Red staining revealed a significant reduction in fibrosis levels in the myocardium of YOD1CKO mice with myocardial infarction (fig. S9, I to L). These results indicate that inhibiting YOD1 can also alleviate pathological cardiac hypertrophy and dysfunction induced by myocardial infarction.

### YOD1 directly interacts with STAT3

**Fig. 4.**
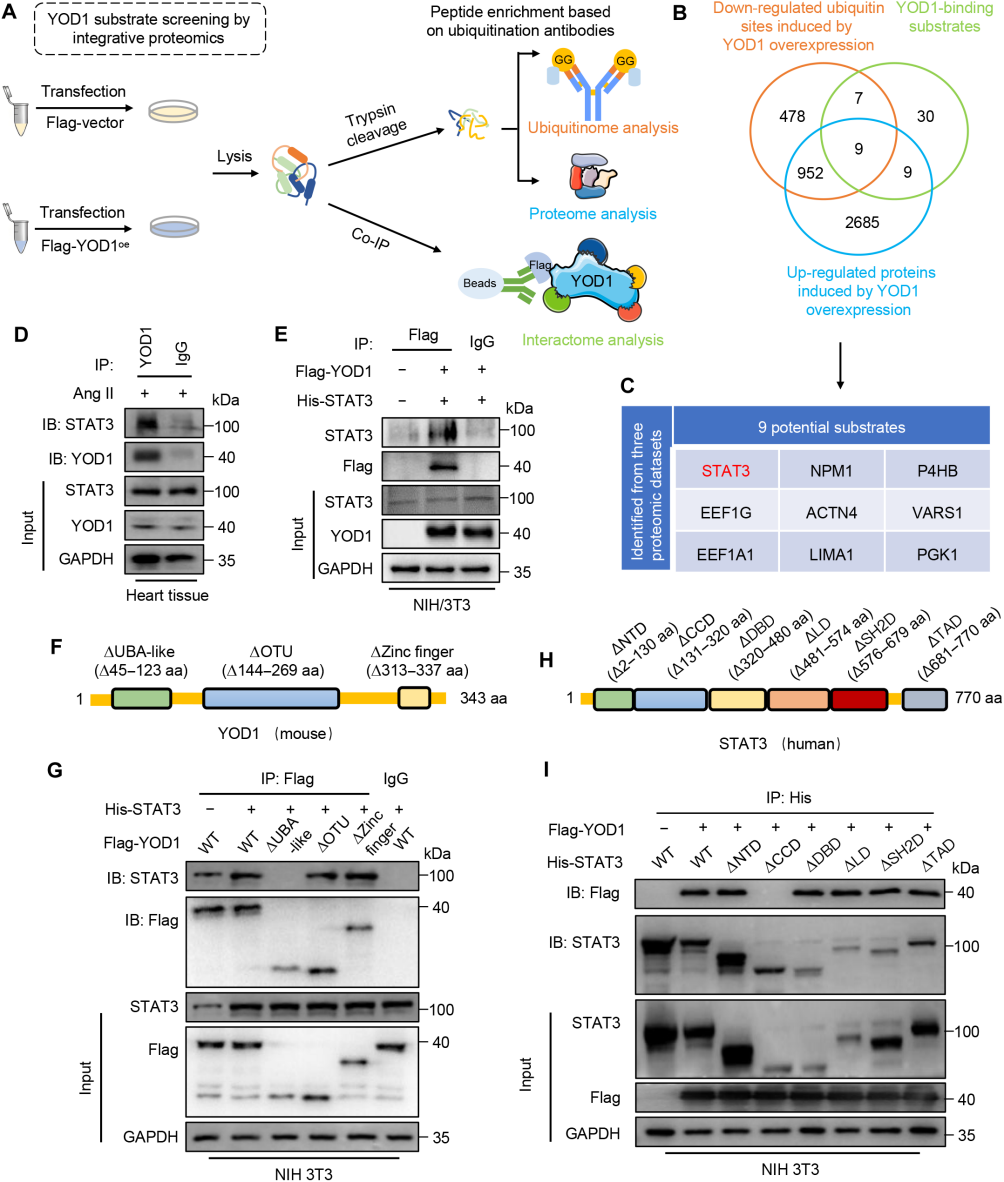
YOD1 directly interacts with STAT3. (**A**) Schematic diagram of three omic processes for YOD1 substrate screening. Cardiomyocytes were transfected with Flag-vector or Flag-YOD1 plasmids, followed by Ang II stimulation (1 μM for 24 hours). Anti-Flag and protein G–Sepharose beads were added to the cell samples for interactome analysis using co-IP. The binding proteins were extracted, digested to peptides, and then subjected to LC-MS/MS measurement for ubiquitinome and proteome analyses. (**B** and **C**) The Venn diagram depicts the integration of three omic approaches for identifying substrate proteins of YOD1 (B). The following table presents the nine candidate substrates of YOD1 that were screened using these three omic methods (C). (**D**) Co-IP of endogenous YOD1 and STAT3 in primary cardiomyocytes following Ang II stimulation. Endogenous YOD1 was immunoprecipitated using an anti-YOD1 antibody. IgG, immunoglobulin G. (**E**) Co-IP of YOD1 and STAT3 in NIH 3T3 cells cotransfected with Flag-YOD1 and His-STAT3 plasmids. Exogenous STAT3 was immunoprecipitated by anti-His antibody. (**F**) A schematic representation of the STAT3 domain deletion construct used in (**G**). aa, amino acids; UBA, Ub-associated domain. (G) Co-IP of WT-STAT3, mut-STAT3, and YOD1 in NIH 3T3 cells cotransfected with overexpression plasmids of Flag-WT-STAT3, Flag-mut-STAT3, and Flag-YOD1. Exogenous normal or mutated STAT3 was immunoprecipitated by anti-His antibody. (**H**) A schematic representation of the YOD1 domain deletion construct used in Fig. 5I. (**I**) Co-IP of WT-YOD1, mut-YOD1, and STAT3 in NIH 3T3 cells cotransfected with overexpression plasmids of Flag-WT-YOD1, Flag-mut-YOD1, and His-STAT3. Exogenous normal or mutated YOD1 was immunoprecipitated by anti-Flag antibody. IB, immunoblotting; NTD, N-terminal Domain; CCD, Coiled-coil Domain; DBD, DNA-binding Domain; LD, Linker Domain; SH2, Src Homology 2 Domain; TAD, Transactivation Domain.

To elucidate the deubiquitinating substrate of YOD1 in cardiac hypertrophy, we performed multiple proteomic analyses, including coimmunoprecipitation (co-IP) combined with liquid chromatography–tandem mass spectrometry (LC-MS/MS) analysis (interactome), ubiquitinome analysis, and proteome analysis in cardiomyocytes transfected with YOD1 or control plasmid (Fig. 4A). Interactome analysis identified the YOD1-binding protein, ubiquitinome analysis identified the deubiquitinated proteins induced by YOD1 overexpression (YOD1^oe^), and proteome analysis identified the proteins whose protein levels were changed in YOD1-overexpressed cardiomyocytes (table S5). Venn analysis on the results of three proteomic methods showed the simultaneous presence of nine proteins across these clusters: STAT3, nucleophosmin (NPM1), protein disulfide isomerase (P4HB), elongation factor 1G (EEF1G), α-actinin 4 (ACTN4), valine–tRNA ligase (VARS1), elongation factor 1–α1 (EEF1A1), *Lin*-11 *Isl*-1 *Mec*-3 (LIM domain) and actin-binding protein 1 (LIMA1), and phosphoglycerate kinase 1 (PGK1) (Fig. 4, B and C). Among these nine, only STAT3 has been reported to play an essential role in the progression of pathological cardiac hypertrophy (*16*, *17*). Therefore, we hypothesize that STAT3 serves as a substrate of YOD1 in cardiomyocytes and mediates YOD1’s role in cardiac hypertrophy.

We confirmed the interaction between endogenous YOD1 and STAT3 in Ang II–induced mouse cardiac tissue (Fig. 4D). Exogenous YOD1 and STAT3 interaction was also observed in NIH 3T3 cells transfected with these two plasmids (Fig. 4E). To further investigate the specific regions in the YOD1-STAT3 interaction, plasmids containing various YOD1 mutants were constructed (Fig. 4F). These mutated plasmids and a WT YOD1 plasmid were cotransfected with STAT3 into NIH 3T3 cells, respectively. Co-IP experiments demonstrated that the OTU domain of YOD1 is responsible for its interaction with STAT3 (Fig. 4G). Concurrently, we constructed mutant plasmids for STAT3 domains (Fig. 4H) and confirmed that the CCD domain (Coiled-coil Domain) of STAT3 is responsible for its interaction with YOD1 (Fig. 4I).

### YOD1 regulates the deubiquitination and stability of STAT3

**Fig. 5.**
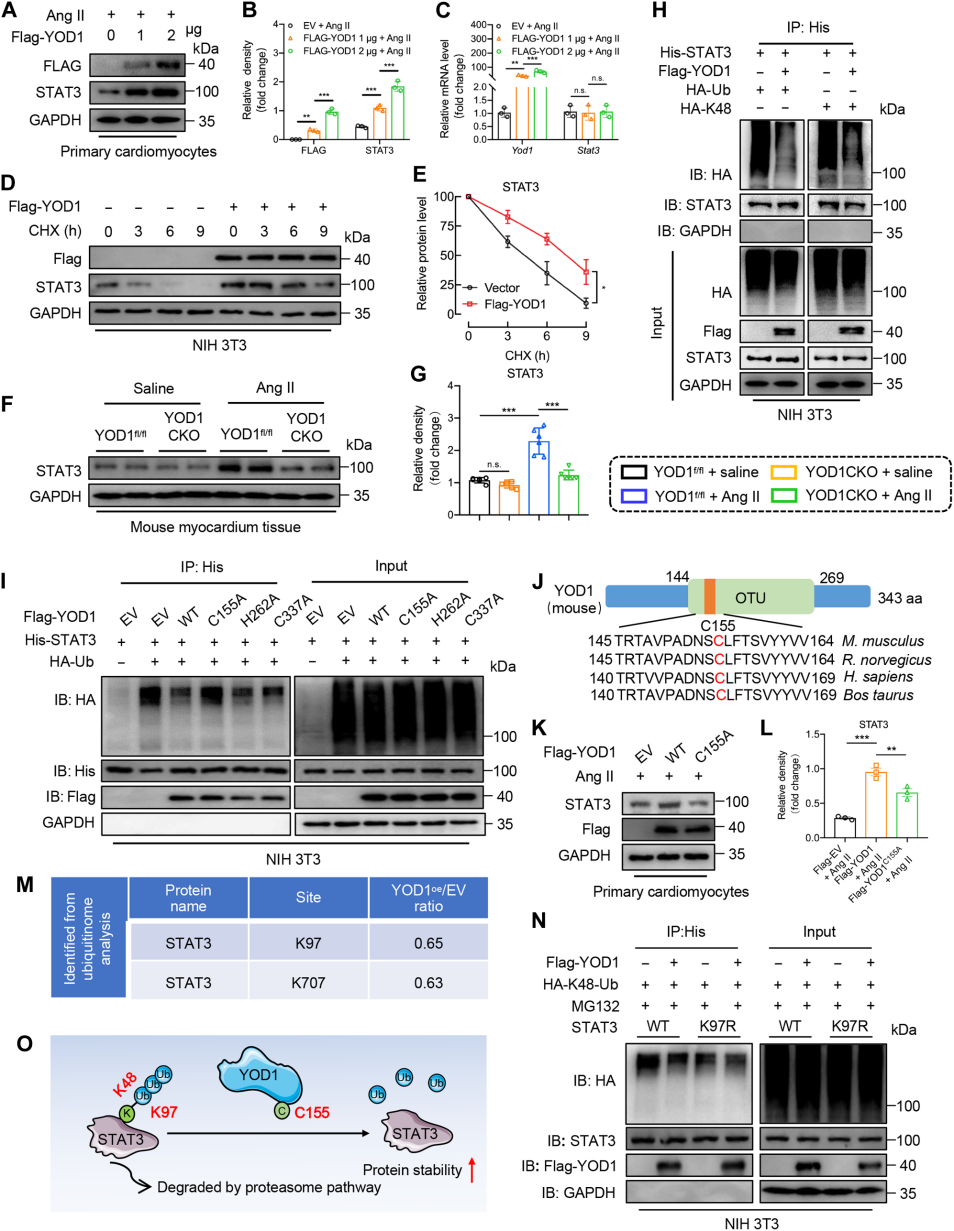
YOD1 regulates the deubiquitination and stability of STAT3. (**A** to **C**) Representative Western blot analysis of YOD1 and STAT3 in primary cardiomyocytes (A) and real-time qPCR analysis of mRNA expression levels of *Yod1* and *Stat3* in NIH 3T3 cells (C) transfected with varying doses of Flag-YOD1^oe^ plasmids and densitometric quantification (B). *n* = 3. (**D** and **E**) Representative Western blot analysis of STAT3 in NIH 3T3 cells transfected with Flag-YOD1^oe^ plasmids, followed by a CHX pulse-chase assay (D) and densitometric quantification (E). *n* = 3. (**F** and **G**) Representative Western blot analysis of STAT3 in Ang II–induced heart tissues from YOD1CKO mice (F) and densitometric quantification (G). *n* = 6. (**H**) Co-IP of STAT3 in NIH 3T3 cells cotransfected with overexpression plasmids for His-STAT3, Flag-YOD1, hemagglutinin (HA)–Ub, and HA-Ub-K48. Ubiquitinated STAT3 was identified to elucidate the ubiquitination pattern of STAT3 regulated by YOD1. (**I**) Co-IP of STAT3 in NIH 3T3 cells cotransfected with overexpression plasmids for Flag-YOD1-WT, Flag-YOD1-Mut, His-STAT3, and HA-Ub. Ubiquitinated STAT3 was detected to elucidate the ubiquitination level of STAT3 regulated by the active site of YOD1. (**J**) Schematic representation of the sequence of YOD1-C155 across various species. (**K** and **L**) Representative Western blot analysis of STAT3 in primary cardiomyocytes transfected with Flag-YOD1-WT and Flag-YOD1-C155A overexpression plasmids (K) and densitometric quantification (L). *n* = 3. (**M**) Ubiquitinome analysis showed YOD1-regulated ubiquitination lysine residues of STAT3. (**N**) Co-IP of STAT3 in NIH 3T3 cells that were cotransfected with overexpression plasmids encoding Flag-YOD1, His-STAT3-WT, His-STAT3-K97R, and HA-Ub-K48. Ubiquitinated STAT3 was detected to elucidate the ubiquitination lysine residues of STAT3 regulated by YOD1. (**O**) Schematic illustrating that YOD1 preserves the stability of STAT3 by deubiquitinating it at residue K97 through its active site C155. n.s., *P* > 0.05; **P < 0.01; ***P < 0.001.

We aim to determine whether YOD1 regulates the protein level of STAT3. We transfected the YOD1^oe^ plasmid in neonatal rat cardiomyocytes (NRCMs) and observed a dose-dependent increase in the protein levels of STAT3 (Fig. 5, A and B). YOD1-increased STAT3 protein level was also accompanied by elevated levels of phosphorylated STAT3 (p-STAT3)^Y705^ and p-STAT3^S727^ in cardiomyocytes (fig. S10, A and B). Notably, this increase in STAT3 protein was not correlated with its mRNA level change (Fig. 5C). Subsequently, we confirmed that YOD1 reduced STAT3 degradation in a time-dependent manner when protein synthesis was inhibited by cycloheximide (CHX) (Fig. 5, D and E). We also observed the reduced levels of total STAT3 and p-STAT3^Y705/S727^ in the myocardial tissue of Ang II–challenged YOD1CKO mice (Fig. 5, F and G, and fig. S11, A and B). These data indicate that YOD1 positively regulates the posttranslational stability of STAT3 protein.

It has been reported that DUBs can inhibit the degradation of substrate proteins by cleaving the K48-linked Ub chain on these proteins (*18*). We then found that YOD1 substantially diminished the ubiquitination of STAT3 protein, and this reduction primarily pertains to Ub molecules that exhibit K48 linkage activity (Fig. 5H). We further investigated the active site of YOD1 mediating STAT3 deubiquitination. Three residues, C155, H262, and C337, have been identified as potential active sites for the enzymatic activity of YOD1. We found that, although YOD1-C155A, YOD1-H262A, and YOD1-C337A mutants are capable of binding to STAT3 (fig. S12), only YOD1-C155A mutant plasmid transfection resulted in the loss of the capacity to deubiquitinate STAT3 (Fig. 5I). The residue C155 in YOD1 is highly conserved across various species (Fig. 5J). Subsequently, we observed that the WT YOD1 plasmid was able to enhance the protein content of STAT3 in Ang II–stimulated cardiomyocytes, while the YOD1-C155A plasmid failed (Fig. 5, K and L). Further analysis of ubiquitomic data highlighted that YOD1 effectively reduced ubiquitination at both residues K97 and K707 on STAT3 protein (Fig. 5M). However, we found that K97R mutant showed stronger ability than K707R mutant to inhibit Ang II–induced ANP and MyHC mRNA levels in cardiomyocytes (fig. S13), indicating that STAT3 deubiquitination at the K97 site is more important for regulating cardiomyocyte hypertrophy. We then showed that the STAT3-K97R mutant no longer deubiquitinated STAT3 in NIH 3T3 cells (Fig. 5N). These findings indicate that the C155 active site of YOD1 is crucial for maintaining the stability of STAT3 by deubiquitinating K48-linked Ub molecules from the K97 site of STAT3 protein (Fig. 5O).

### YOD1 facilitates the nuclear translocation of STAT3

**Fig. 6.**
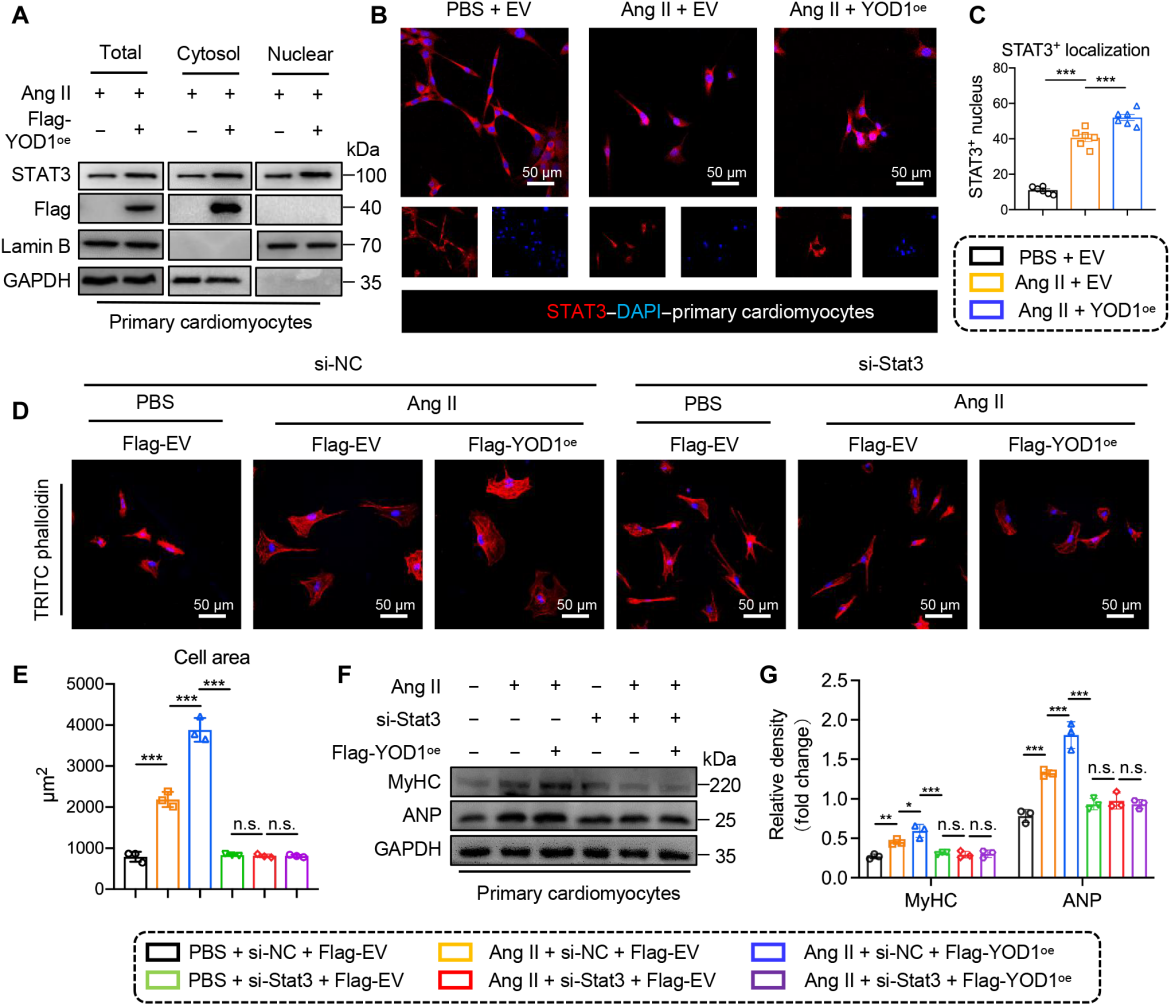
YOD1 facilitates the nuclear translocation of STAT3. Cardiomyocytes from (A) to (C) were transfected with plasmids containing either the empty vector or YOD1 (YOD1^oe^), followed by stimulation with Ang II (1 μM for 24 hours). Cardiomyocytes from (D) to (G) were transfected with plasmids containing either the empty vector or YOD1 (YOD1^oe^) and siRNAs targeting negative control or STAT3, followed by stimulation with Ang II (1 μM for 24 hours). (**A**) Representative Western blot analysis of STAT3 in total cell lysate, cytoplasmic lysate, and nuclear lysate. Glyceraldehyde phosphate dehydrogenase (GAPDH) and lamin B were used as loading controls. (**B** and **C**) Representative images of immunofluorescence staining illustrating STAT3 nuclear translocation in cardiomyocytes (B), along with the corresponding quantitative analysis (C). The staining results are presented as follows: Red represents STAT3, while blue indicates 4′,6-diamidino-2-phenylindole (DAPI). Scale bars, 50 μm. *n* = 6. (**D** and **E**) The surface area of the cardiomyocytes was assessed using TRITC-labeled rhodamine-phalloidin staining (D), accompanied by a corresponding quantitative analysis (E). *n* = 3. (**F** and **G**) Representative Western blot analysis of MyHC and ANP in cardiomyocytes (F) and densitometric quantification (G). n.s., *P* > 0.05; *P < 0.05; **P < 0.01; ***P < 0.001.

Upon its translocation to the nucleus, STAT3 binds to specific promoters, leading to the transcription of target genes that facilitate cardiac hypertrophy (*19*). Initially, we observed that overexpression of YOD1 in Ang II–stimulated cardiomyocytes enhanced the level of STAT3 in the nucleus, and this increase predominantly resulted from an overall elevation of STAT3 protein (Fig. 6, A to C). Previous studies have demonstrated that the inhibition of STAT3 signaling, either through Stattic or siRNA knockdown, can significantly ameliorate cardiac hypertrophy (*20*–*22*). In the present study, tetramethyl rhodamine isothiocyanate (TRITC)–phalloidin staining showed that YOD1 overexpression significantly exacerbated Ang II–induced cardiomyocyte hypertrophy. Conversely, the reduction of STAT3 expression via siRNA leads to a decrease in Ang II–induced cardiomyocyte hypertrophy. Notably, knockdown of STAT3 in Ang II–stimulated cardiomyocytes effectively offsets the cardiomyocyte hypertrophy induced by YOD1 overexpression (Fig. 6, D and E, and fig. S14). Likewise, protein levels of MyHC and ANP in cardiomyocytes exhibited a similar trend (Fig. 6, F and G). These findings indicate that YOD1 promotes cardiac hypertrophy by elevating the protein levels of STAT3 in cardiomyocytes.

### YOD1 enhances Ang II–induced cardiac hypertrophy dependent on STAT3

**Fig. 7.**
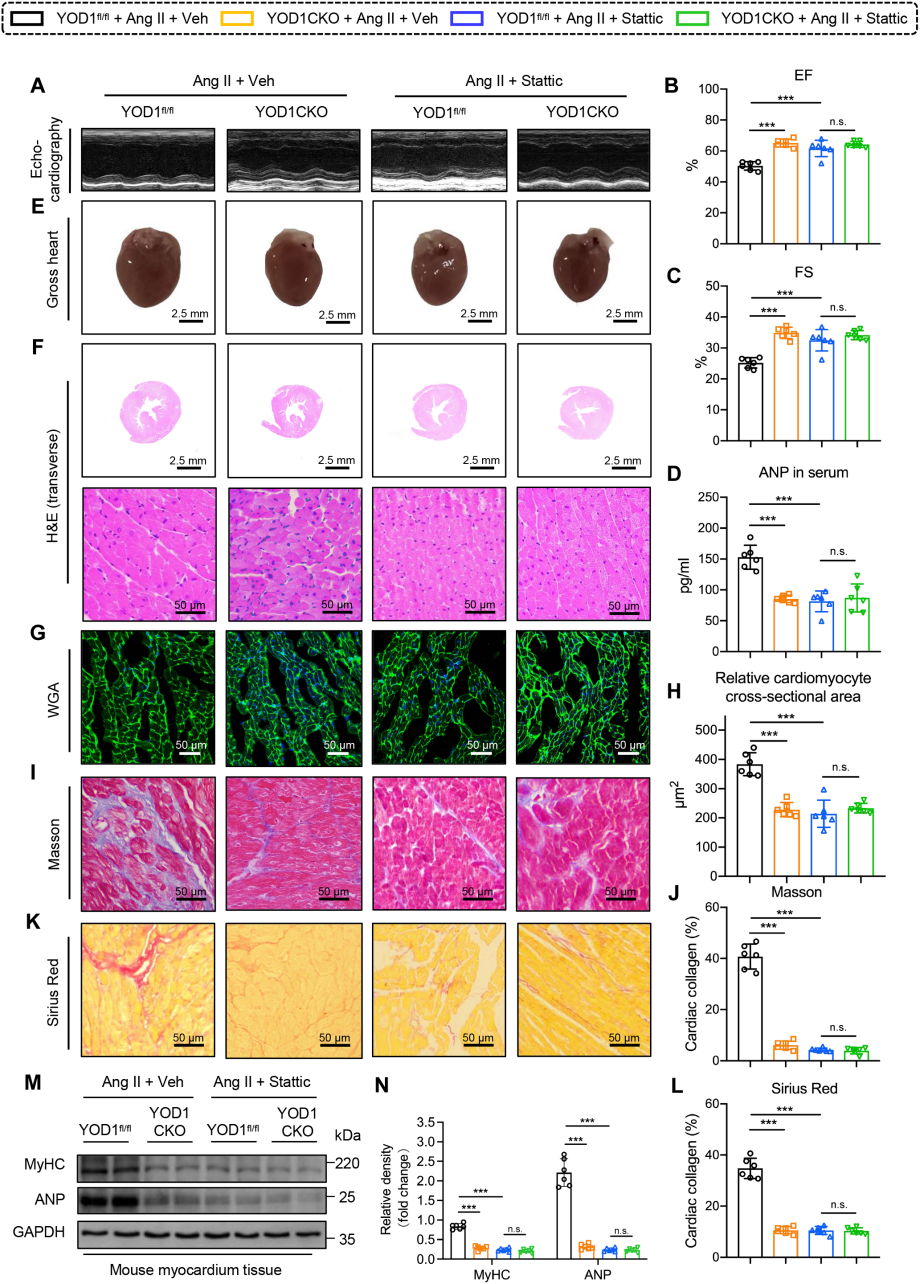
YOD1 enhances Ang II–induced cardiac hypertrophy dependent on STAT3. To verify the effect of YOD1 on STAT3, the STAT3 inhibitor Stattic (10 mg/kg, gavage) or vehicle [0.5% *O*-carboxymethylcellulose (CMC)–Na and 0.25% Tween 80] was administered every 3 days via oral gavage. Healthy male YOD1CKO mice aged 6 to 8 weeks and YOD1^fl/fl^ mice were injected with Ang II (1 μg kg^–1^ min^–1^) or normal saline via an osmotic pump (catalog no. ALZET Model 1004, USA) for 4 weeks to induce cardiac hypertrophy. (**A**) Representative M-mode echocardiography of mice in each group. (**B** and **C**) Myocardial function parameters, including EF (B) and FS (C), were evaluated in mice through echocardiography. (**D**) The plasma levels of ANP in each group. (**E**) Representative images of whole hearts. Scale bars, 2.5 mm. (**F**) Representative images of H&E staining of transverse section in myocardium tissues. Scale bars, 2.5 mm and 50 μm. (**G** and **H**) Representative images of WGA (G) staining in sections of hearts and quantitative area analysis (H). Scale bars, 50 μm. (**I** to **L**) Fibrotic areas were evaluated using Masson’s trichrome (I) and Sirius Red (K) staining, as well as the quantification of fibrotic regions (H and J) in heart sections from each group. Scale bars, 50 μm. (**M** and **N**) Representative Western blot analysis of MyHC and ANP (M) and densitometric quantification (N). *n* = 6. n.s., *P* > 0.05; ***P < 0.001.

We treated Ang II–infused YOD1^fl/fl^ and YOD1CKO mice with Stattic, a specific STAT3 inhibitor. As demonstrated in Fig. 7 (A to C) and table S6, either YOD1CKO or stattic treatment significantly ameliorated Ang II–induced cardiac dysfunction. However, the addition of stattic to YOD1CKO mice did not yield further improvement in cardiac function compared to that in stattic-treated YOD1^fl/fl^ mice. In addition, YOD1CKO did not provide additional benefits against Ang II–induced cardiac hypertrophy in stattic-treated mice (Fig. 7, D to H, fig. S15A, and table S6). Masson’s trichrome and Sirius Red staining revealed that YOD1CKO did not enhance protection against Ang II–induced cardiac fibrosis post–STAT3 inhibition (Fig. 7, I to L, and fig. S15, B to E). A consistent trend was observed in the changing profiles of MyHC, ANP, Col-I, and TGF-β in mouse heart tissues (Fig. 7, M and N, and fig. S15, F and G). These outcomes indicate that the YOD1-STAT3 axis plays a significant role in regulating pathological cardiac remodeling induced by Ang II.

### Pharmacological inhibition of YOD1 alleviates Ang II–induced cardiac hypertrophy

**Fig. 8.**
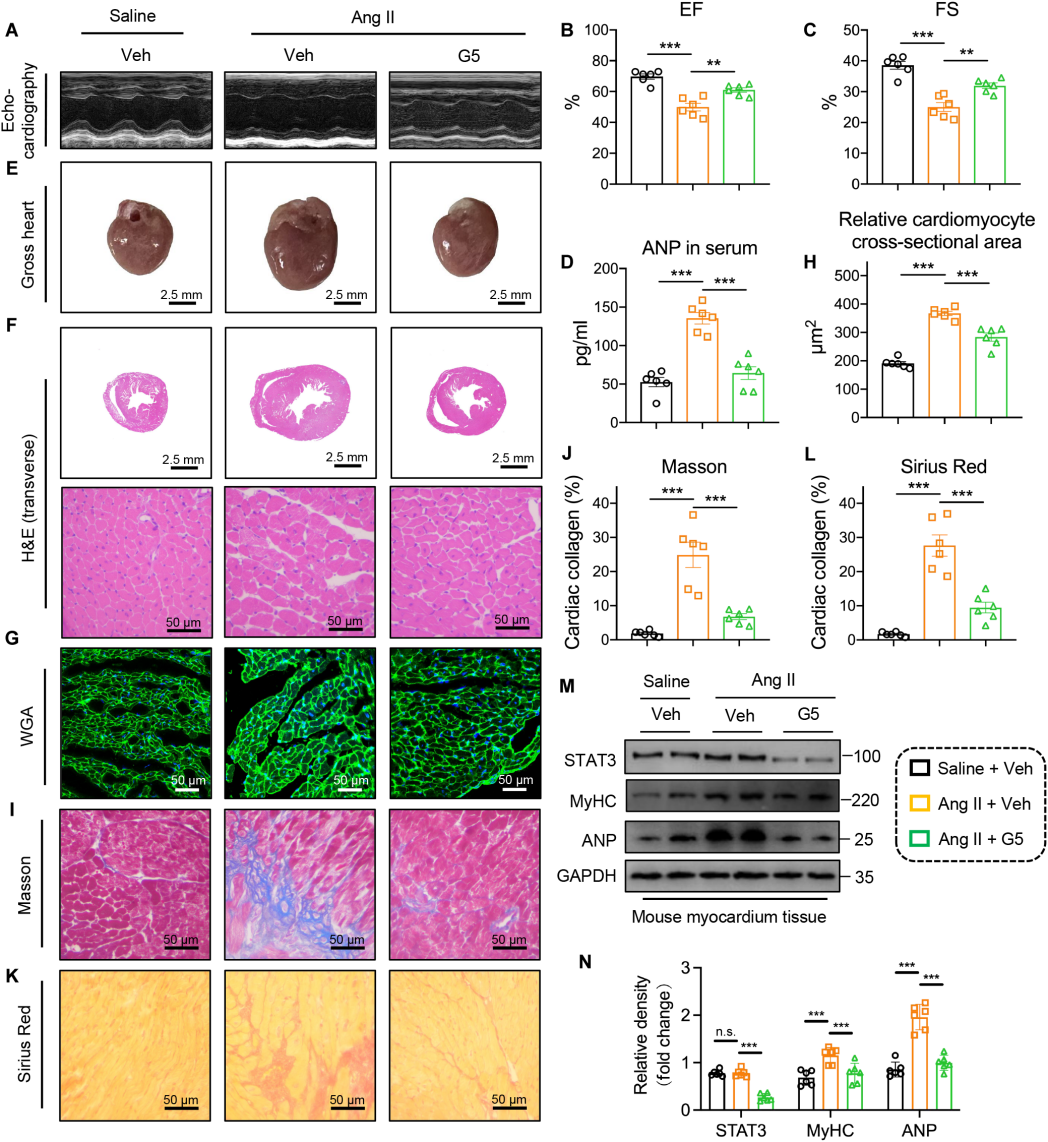
Pharmacological inhibition of YOD1 alleviates Ang II–induced cardiac hypertrophy. To investigate the pharmacological inhibition of YOD1 on cardiac hypertrophy, the YOD1 inhibitor G5 (10 mg/kg, gavage) or vehicle (0.5% CMC-Na and 0.25% Tween 80) was delivered every 3 days using oral gavage. Healthy male WT mice aged 6 to 8 weeks were injected with Ang II (1 μg kg^–1^ min^–1^) or normal saline via an osmotic pump (catalog no. ALZET Model 1004, USA) for 4 weeks to induce cardiac hypertrophy. (**A**) Representative M-mode echocardiography of mice in each group. (**B** and **C**) Myocardial function parameters, including EF (B) and FS (C), were evaluated in mice through echocardiography. (**D**) The plasma levels of ANP in each group. (**E**) Representative images of whole hearts. Scale bars, 2.5 mm. (**F**) Representative images of H&E staining of transverse section in myocardium tissues. Scale bars, 2.5 mm and 50 μm. (**G** and **H**) Representative images of WGA (G) staining in sections of hearts and quantitative area analysis (H). Scale bars, 50 μm. (**I** to **L**) Fibrotic areas were evaluated using Masson’s trichrome (I) and Sirius Red (K) staining, as well as the quantification of fibrotic regions (H and J) in heart sections from each group. Scale bars, 50 μm. (**M** and **N**) Representative Western blot analysis of STAT3, MyHC, and ANP (M) and densitometric quantification (N). *n* = 6. n.s., *P* > 0.05; **P < 0.01; ***P < 0.001.

Last, we examined the effects of pharmacological inhibition of YOD1. The small molecule G5 has been reported to pharmacologically inhibit YOD1 and consequently exert antiacute promyelocytic leukemia effects (*9*). Therefore, we aimed to evaluate whether G5 also has antihypertrophic properties. As expected, G5 treatment significantly improved cardiac function and hypertrophy in Ang II–challenged mice (Fig. 8, A to H, fig. S16A, and table S7). Masson’s trichrome and Sirius Red staining confirmed that G5 reduced Ang II–induced cardiac fibrosis (Fig. 8, I to L, and fig. S16, B to E). Similar results were observed when we examined the protein levels of MyHC, ANP, Col-I, and TGF-β in mouse hearts. At the same time, the results revealed that G5 significantly reduced the protein level of STAT3 in the myocardial tissue of Ang II–infused mice (Fig. 8, M and N, and fig. S16, F and G). These findings suggest that pharmacologically inhibiting YOD1 improves Ang II–induced cardiac remodeling and dysfunction.

## DISCUSSION

In this study, we observed that YOD1 was overexpressed in cardiomyocytes during pathological cardiac hypertrophy. We confirmed that knockout of YOD1 specifically in cardiomyocytes effectively alleviated Ang II– and TAC-induced cardiac hypertrophy. Subsequently, we used ubiquitination omics, protein interaction omics, and proteomics to identify STAT3 as the substrate protein of YOD1. In terms of mechanism, we revealed that the C155 active site of YOD1 removes the K48-linked Ub chain at the K97 residue of STAT3 protein, thereby increasing the stability of intracellular STAT3 levels. Inhibition of STAT3 abolished the cardioprotective effects of YOD1CKO in Ang II–infused mice, validating the YOD1-STAT3 axis in regulating cardiac hypertrophy. Moreover, treatment with a pharmacological inhibitor of YOD1 significantly ameliorates Ang II–induced cardiac dysfunction in mice. This study elucidates the role of YOD1 in pathological cardiac hypertrophy.

There are more than 110 DUBs, classified into seven families. Current research primarily focuses on the Ub-Specific Protease (USP) family, specifically the regulatory roles of USP7 (*23*), USP14 (*24*), USP18 (*25*), USP19 (*26*), and USP38 (*27*) in cardiac hypertrophy. Our team has previously demonstrated that cardiomyocyte USP25 exerts an antihypertrophy effect through its regulation of Sarco/Endoplasmic Reticulum Calcium ATPase 2a (SERCA2a)–mediated sarcoplasmic reticulum calcium transport (*28*). We also revealed that cardiomyocyte-derived USP28 negatively regulates antioxidant responses and promotes cardiac hypertrophy by deubiquitinating Tripartite Motif Containing 21 (TRIM21) (*29*). Recently, a few studies suggest that DUBs in the OTU family also participate in pathological cardiac hypertrophy. For example, OTUD6a exacerbates cardiac inflammation and cardiac hypertrophy via deubiquitinating Stimulator of Interferon Genes (STING) (*30*), and cardiomyocyte OTUD1 promotes heart failure by regulating deubiquitination of STAT3 (*12*, *31*). This study confirmed that YOD1 exhibits a notable increase in both Ang II– and TAC-induced mouse hearts. We demonstrate the role and regulatory mechanism of cardiomyocyte YOD1, a DUB in the OTU family, in cardiac hypertrophy.

Numerous studies have confirmed the crucial role of STAT3 in pathological cardiac hypertrophy (*16*). As a DNA binding transcriptional activator, STAT3 directly binds to hypertrophic and profibrotic gene promoters to up-regulate the transcription and expression of proteins related to cardiac remodeling, such as MyHC, ANP, Col-I, and TGF-β in cardiomyocytes (*21*, *22*). Therapeutic strategies targeting STAT3 to treat cardiac hypertrophy have recently emerged (*32*). Our team has reported the cardioprotective effects of natural product celastrol via directly targeting STAT3 (*22*). However, targeting STAT3 for the treatment of heart disease remains very challenging. A big concern is that STAT3 is widely expressed across various tissues and regulates multiple pathophysiological processes (*19*). The non–tissue-targeted STAT3 inhibitors may induce potential side effects in other systems. Therefore, identifying upstream and specific targets for inhibiting STAT3 may represent a more effective approach. YOD1 is mainly expressed in hypertrophic cardiomyocytes under pathological conditions and functions by specifically targeting STAT3 in cardiomyocytes. Therefore, selecting YOD1 as an intervention on the YOD1-STAT3 axis can specifically inhibit STAT3 in pathological hypertrophic hearts, with less impacts on other tissues or cells.

Recent studies have indicated that the ubiquitination modification of STAT3 plays a significant role in various biological processes. Both K48- and K63-linked ubiquitination were observed with STAT3 in the spinal cords of aging mice, and these two ubiquitinating forms of STAT3 exhibited considerable increases in aged spinal cord tissue (*33*). K48 Ub chain modifications are primarily responsible for mediating substrate degradation, whereas K63-linked ubiquitination facilitates function or interaction of substrate proteins (*34*). E3 Ub ligases such as Synoviolin 1 (SYVN1) (*35*), Cullin-associated and neddylation-dissociated 1 (CAND1) (*36*), Constitutive Photomorphogenic 1 (COP1) (*37*), and PDZ and LIM domain protein 2 (PDLIM2) (*38*) have been reported to ubiquitinate STAT3 to promote its degradation via the Ub-proteasome pathway. In addition, tumor necrosis factor receptor–associated factor 6 has been shown to add K63-linked polyubiquitin chains to STAT3, which inhibits STAT3 activation (*39*). Our group also previously demonstrated that OTUD1 (*12*) and OTUD6A (*40*) could deubiquitinate STAT3 in a K63-linked way, which subsequently inhibited STAT3 phosphorylation activation. Other groups have reported that USP7 (*41*) and USP28 (*42*) enhance the stability of STAT3 by mediating its deubiquitination. However, none of these studies reported the specific ubiquitination sites on STAT3 protein. Identifying these ubiquitination/deubiquitination modification sites is crucial for future development of drugs targeting STAT3. In this study, we demonstrate that the K48-linked Ub chain at the K97 site of STAT3 can be removed by YOD1. Notably, our data show that neither the overexpression nor deletion of YOD1 affected the ratio of p-STAT3 to total STAT3, suggesting that while YOD1 influences the overall content of STAT3, it does not directly affect its phosphorylation activity. The current study paves the way for further elucidation of the ubiquitination landscape of STAT3 and enhances our understanding of how these ubiquitinating modifications regulate STAT3 protein function.

So far, only one compound G5 has been reported to inhibit YOD1 as an inhibitor of YOD1 (*43*). We also found that pharmacological inhibition of YOD1 by G5 prevented Ang II–induced cardiac remodeling in mice. However, it remains unclear whether G5 specifically targets YOD1. The development of specific small-molecule YOD1 inhibitors represents a worthwhile avenue of investigation. In this study, we determined that the C155 site of YOD1 functions as an active center for STAT3 deubiquitination. This active site lays an experimental foundation for designing small molecules inhibiting YOD1. Protein-protein interactions (PPIs) are crucial in biological processes and are increasingly recognized as promising targets for therapeutic interventions (*44*). Here, we demonstrate that the OTU domain of YOD1 directly interacts with the CDD domain of STAT3. As a result, designing the PPI inhibitors targeting the interaction interface between YOD1 and STAT3 may be interesting and deserves further investigation.

Admittedly, several substrates of YOD1 have been revealed in our three proteomic analyses, and we cannot completely exclude the possibility that YOD1 regulates the cardiomyocyte hypertrophic response through substrates in addition to STAT3. Collectively, we showed that cardiomyocyte YOD1 deubiquitinates and stabilizes STAT3 to drive cardiac hypertrophy, fibrosis, and dysfunction. We also demonstrated the protective role of cardiomyocyte-specific knockout and pharmacological inhibition of YOD1 against myocardial hypertrophy, suggesting that cardiac-specific therapy targeting YOD1 may be an attractive strategy for the treatment of cardiac hypertrophy.

## MATERIALS AND METHODS

### Reagents

Ang II (catalog no. HY-13948), Stattic (catalog no. HY-13818), and CHX (catalog no. HY-12320) were obtained from MedChemExpress (New Jersey, USA). The YOD1 inhibitor G5 (catalog no. BD159874) was purchased from Bidd Pharmaceutical Technology (Shanghai, China). siRNA targeting STAT3 and YOD1 was sourced from RiboBio (Guangzhou, China). Plasmids, including Flag-YOD1, His-STAT3, hemagglutinin (HA)–Ub, HA-Ub-K48, Flag-YOD1-mutant, and His-STAT3-mutant, were acquired from GeneChem (Shanghai, China). Antibodies against YOD1 (catalog no. A13270, RRID: AB_2760122; dilution: 1:1000) were obtained from ABclonal Technology (Wuhan, China). Antibodies targeting glyceraldehyde phosphate dehydrogenase (catalog no. 5174, RRID: AB_10622025; dilution: 1:1000) and vimentin (catalog no. 5741S, RRID: AB_10695459; dilution: 1:200) were procured from Cell Signaling Technology (CST; MA, USA). In addition, antibodies against MyHC (catalog no. ab50967, RRID: AB_942084; dilution: 1:1000), TGF-β (catalog no. ab179695, RRID: AB_2938687; dilution: 1:1000), Col-I (catalog no. ab270993, RRID: AB_2927551; dilution: 1:1000), lamin B (catalog no. ab16048, RRID: AB_443298; dilution 1:1000), and CD68 (catalog no. ab283654, RRID: AB_2922954; dilution: 1:200) were acquired from Abcam (Cambridge, UK). Immunoglobulin G (catalog no. B900610, RRID: AB_3674206) and antibodies against Flag (catalog no. 20543-1-AP, RRID: AB_11232216; dilution: 1:1000), HA (catalog no. 51064-2-AP, RRID: AB_11042321; dilution: 1:1000), and His (catalog no. 66005-1-IG, RRID: AB_11232599; dilution: 1:10,000) were procured from Proteintech (Hubei, China). Antibodies targeting ANP (catalog no. SC-515701, RRID: AB_3076640; dilution: 1:1000) were sourced from Santa Cruz Biotechnology (Texas, USA). The antibody against α-actin (catalog no. ET1607-53-50ul, RRID: AB_3069772; dilution: 1:200) and STAT3 (catalog no. ET1607-38-50ul, RRID: AB_3069762; dilution: 1:1000) was obtained from HuaBio (Hangzhou, China). The H&E staining kit (catalog no. G1120), the Masson’s trichrome staining kit (catalog no. G1340), and the Sirius Red staining kit (catalog no. S8060) were acquired from Solarbio (Beijing, China).

### Human heart samples

Hypertrophic myocardium samples were acquired from patients diagnosed with heart failure, as detailed in a prior study (*45*). All experiments involving human subjects received approval from the Ethics Committee of The First Affiliated Hospital of Wenzhou Medical University (Wenzhou, China; approval no. KY2022-156) and complied with the principles outlined in the Declaration of Helsinki. The clinical characteristics of the patients are presented in table S8. Written informed consent was obtained from all study participants.

### Animal experiment

The experimental procedures and animal rearing conditions were approved by the Experimental Animal Ethics Committee at Wenzhou Medical University (WYYY-AEC-YS-2024-0190). All animal studies adhered to the review and approval of nurturing and experimental protocols granted by the Animal Policy and Welfare Committee of Wenzhou Medical University. These practices comply with the guidelines outlined in the Guide for the Care and Use of Laboratory Animals, published by the National Institutes of Health in the United States. All animals originated from a single litter. Grouping was conducted using a randomized method, while experiments and analyses were performed by blinded experimenters. The YOD1^−/−^ mice were purchased from Shanghai Model Organisms Center. In addition, YOD1^fl/fl^Myh6-Cre (YOD1CKO) mice were obtained via crossing YOD1^fl/fl^ with Myh6 (Myosin Heavy Chain 6)–Cre mice.

To establish a model of Ang II–induced cardiac hypertrophy, healthy male mice aged 6 to 8 weeks were administered Ang II (1 μg kg^–1^ min^–1^) or normal saline via an osmotic pump (catalog no. ALZET model 1004, USA) for a duration of 4 weeks, according to our previous research findings (*12*). Briefly, the hair on the lower back of the mice was meticulously removed, and their skin was thoroughly cleansed with an iodophor solution. A precise incision was made to facilitate the insertion of a micropump while minimizing damage to surrounding tissues. The peritoneal cavity was gently expanded, and the micropump containing Ang II (at a dosage of 1 μg kg^–1^ min^–1^) was carefully inserted. Last, meticulous suturing closed the wound, and disinfection with iodophor solution ensured sterility. During the first week postsurgery, daily observations were conducted on each mouse’s incision site, while blood pressure and body weight measurements were recorded every 3 days. The success of the model was determined by systolic blood pressure exceeding 150 mmHg and remaining elevated for a sustained period of 4 weeks. Four weeks following surgery, mice were euthanized to collect serum and heart tissue samples.

For the model of cardiac hypertrophy induced by TAC, healthy male mice aged 6 to 8 weeks were selected and anesthetized with isoflurane using an anesthesia machine, in accordance with our previous research (*28*). Briefly, the mice underwent depilation of their neck and chest fur. The skin on the neck was disinfected and incised to expose the trachea, through which a tracheal tube was inserted via the oral cavity. This tube was then connected to a ventilator. An incision was made along the sternum at the level of the second rib to create an open wound, allowing for complete exposure of both the upper edge of the aortic arch and the left common carotid artery. Suturing techniques were used to constrict the aortic arch accordingly. In contrast, for the sham group, sutures were placed but not ligated at this active site. During TAC surgery, there was a mortality rate of 20%, leading to exclusion of these deceased mice from further analysis.

For the myocardial infarction–induced cardiac remodeling model, healthy male mice aged 6 to 8 weeks were selected and anesthetized with isoflurane using an anesthesia machine. Following coronary artery ligation for a duration of 4 weeks, the mice were euthanized under sodium pentobarbital anesthesia after assessing cardiac function via noninvasive echocardiography (FUJIFILM VisualSonics, Canada). Serum and heart tissue from both infarcted and noninfarcted areas were collected for subsequent analyses.

The STAT3 inhibitor Stattic (10 mg/kg) and the YOD1 inhibitor G5 (10 mg/kg) were administered orally every 4 days concurrently with Ang II treatment until the completion of the modeling phase. We used a 0.5% *O*-carboxymethylcellulose (CMC)–Na solution containing 0.25% Tween 80 to dissolve Stattic and G5, ensuring that the solute control group received an equivalent volume of CMC-Na as was provided to the control group.

### Echocardiography

The mice, anesthetized with isoflurane, were carefully positioned on a platform and connected to a noninvasive mechanical ventilation system. The multimode small animal ultrasound imaging system (Vevo 3100, FUJIFILM VisualSonics, Canada) was used to acquire the short-axis section indices.

### Enzyme-linked immunosorbent assay

The concentrations of ANP (catalog no. F10062, Xitang Biotechnology, Shanghai, China) in the serum samples of mice were quantified using enzyme-linked immunosorbent assay kits.

### Histological analysis

The myocardial tissue was fixed using a 4% formaldehyde fixative solution (catalog no. BL539A, Biosharp) and subsequently embedded in paraffin for preservation. Thin sections of the paraffin-embedded tissues were then prepared and stained with H&E staining (catalog no. G1120, Solarbio) to visualize cardiac hypertrophy. In addition, Masson’s trichrome staining and Sirius Red staining were applied to these sections to evaluate the levels of fibrosis within the heart tissue.

For further analysis, the myocardial tissue was preserved using optimal cutting temperature compound (catalog no. 4583, Biosharp, Shanghai, China) before sectioning. The resulting slices were then subjected to WGA–fluorescein isothiocyanate staining (catalog no. GTX01502, GeneTex, Texas, USA) following the manufacturer’s instructions.

### Cell culture and transfection

Neonatal rat primary cardiomyocytes were isolated from the ventricles of neonatal Sprague-Dawley rats. It is essential to sterilize the instruments and prepare an enzymatic solution for the digestion of myocardial tissue. The formulation for 200 ml of solution includes the following: 0.16 g of trypsin (catalog no. T8150, Solarbio), 1.6 g of NaCl (catalog no. 7647-14-5, Merck Millipore), 0.07 g of NaHCO_3_ (catalog no. S837271-25g, MACKLIN), 0.198 g of glucose (catalog no. G8270, Merck Millipore), 0.059 g of KCl (catalog no. P9921, Solarbio), 0.4 g of Hepes (catalog no. H8090, Solarbio), and 0.1 g of Col-II (catalog no. C8150, Solarbio). The entire body of newborn rats aged between 1 and 3 days old was disinfected. A vertical incision along the left margin of the sternum handle was made, followed by an oblique incision toward the right to expose the heart gently. Using tweezers, the heart was cautiously extracted, and any blood residue was washed away in phosphate-buffered saline (PBS). Subsequently, the purified cardiac tissue was transferred into a glass container, and 10 ml of digestive solution was added. The heart was sectioned into smaller fragments, and this mixture was immersed in a constant-temperature magnetic stirrer set at 37°C with an appropriate speed for centrifugation. We discarded the first two supernatants while collecting all subsequent supernatants from this process. We centrifuged all accumulated supernatant samples and supplement them with 5 ml of Dulbecco’s modified Eagle’s medium (DMEM; catalog no. C11995500BT, Gibco) for resuspension before passing through a filter. Last, the filtered supernatant was transferred onto a plate. Because of varying adherence rates between primary cardiomyocytes and fibroblasts, we were able to obtain relatively pure primary cardiomyocytes based on their respective adhesion times. The NIH 3T3 (catalog no. GNM 6, RRID: CVCL_0594) and HL-1 cells (catalog no. iCell-m077, RRID: CVCL_0303) were obtained from the Shanghai Institute of Biochemistry and Cell Biology (Shanghai, China).

These cells were maintained in a humidified incubator at 37°C with 5% CO_2_. The culture medium for these cells comprised DMEM supplemented with 10% fetal bovine serum (catalog no. 10099141C, Gibco) and 1% penicillin/streptomycin (catalog no. BC-CE-007, SenBeiJia Biological Technology).

Upon reaching a cell density of 60% in the six-well plate, we proceeded to transfect the cells with 1 μg of plasmid DNA using Opti-MEM Medium (catalog no. 31985070, Thermo Fisher Scientific, Germany), along with the addition of 2 μl of Lipofectamine 3000 and 2 μl of P3000 (catalog no. L3000-015, Thermo Fisher Scientific, Germany). For siRNA transfection at a concentration of 50 nM, we used Opti-MEM Medium supplemented with 2 μl of Lipofectamine 2000 (catalog no. 11668030, Thermo Fisher Scientific, Germany) as well. Following transfection, the cells were incubated for 24 hours before proceeding to further experimental procedures.

### TRITC-phalloidin staining

Primary cardiomyocytes were cultured in glass-bottom dishes that had been thoroughly cleaned with sterile PBS at 37°C. Following this, the cells were fixed at room temperature for 10 min using a 4% formaldehyde solution. After removing excess formaldehyde with PBS, the cells were permeabilized with a 0.5% Triton X-100 solution for 5 min. Subsequently, primary cardiomyocytes were incubated with TRITC-labeled phalloidin working solution (catalog no. CA1610, Solarbio, Beijing, China) at room temperature in the dark for 30 min. Last, nuclei were stained with 4′,6-diamidino-2-phenylindole (DAPI) solution (catalog no. C0065, Solarbio, Beijing, China), and fluorescence observation was conducted under a fluorescence microscope, followed by photography.

### Immunofluorescence

The prepared frozen sections were immersed in PBS and washed three times on a shaking table, with each wash lasting 5 min. Subsequently, the sections were sealed with a 5% bovine serum albumin (catalog no. A1933, Merck Millipore) solution for 30 min and then incubated overnight with a diluted primary antibody (1:50). After incubation, the frozen sections were washed three times with PBS and then incubated at room temperature for 2 hours with a secondary antibody (1:200). Following the complete removal of the secondary antibody, the sections were sealed using DAPI containing an antifluorescence quencher (catalog no. 0100-20, SouthernBiotech). Once sealing was completed, the sections were examined under a confocal fluorescence microscope (NIS-Elements Viewer) to capture high-quality images.

### Co-IP assay

Two hundred microliters of NP-40 lysis buffer (catalog no. P0013F, Beyotime) was added to the cell samples intended for co-IP experiments after washing with PBS. Once complete lysis is achieved on ice, the samples at 4°C and 12,000 rpm were centrifuged for 10 min to eliminate any sediment at the bottom. The supernatant was divided into two aliquots: one portion of 40 μl as the input sample and another portion of 160 μl as the IP sample. For the input sample, 10 μl of a 5× protein loading buffer (catalog no. FD006, Hangzhou Fude Biological Technology) was added. For the IP sample, 20 μl of washed Protein A + G agarose beads (catalog no. P2012, Beyotime) was incorporated, followed by incubation at 4°C for 1 hour to reduce nonspecific binding. Upon completion of this incubation step, the Protein A + G agarose was removed, and the primary antibody was introduced before allowing it to incubate overnight at 4°C. The subsequent samples should include additional washed Protein A + G agarose and be incubated under identical temperature conditions for 4 hours. After this incubation period, the supernatant was discarded, and the Protein A + G agarose was washed five times with PBS, ensuring that each PBS solution is discarded thereafter. Fifty microliters of diluted (1×) protein loading buffer was added to the agarose beads and incubated in a water bath at 100°C for 10 min. Following this procedure, the agarose beads were extracted from the solution. Last, both input and IP samples were used for Western blot analysis.

### Co-IP combined with LC-MS/MS analysis (interactome analysis)

HL-1 cells were transfected with either Flag-vector or Flag-YOD1 plasmids, followed by stimulation with Ang II. Anti-Flag antibodies and Protein G–Sepharose beads were added to the cell samples for co-IP. The bound proteins were subsequently extracted from the co-IP beads using SDT lysis buffer. Protein digestion to peptides was performed using the Filter-Aided Sample Preparation (FASP) method. Following this, LC-MS/MS analysis was conducted by PTM BIO Co. Ltd. (Zhejiang, China). Candidate substrate proteins were identified on the basis of the intensity of the detected proteins.

### Ubiquitinome and proteome analyses

HL-1 cells were transfected with either Flag-vector or Flag-YOD1 plasmids, followed by stimulation with Ang II. Proteins were subsequently extracted from the HL-1 cells. The peptides were generated by subjecting the proteins to two rounds of trypsin digestion. For ubiquitin omic analysis, the resulting peptides were enriched for Ub peptides by dissolving them in Immunoprecipitation Assisted Purification (IAP) buffer and incubating with anti–K-Ub antibody beads (catalog no. 5562, CST) at 4°C overnight. LC-MS/MS analysis and subsequent ubiquitinome analyses were conducted by Bioprofile (Shanghai, China).

### Western blot analysis

The cells were lysed using radioimmunoprecipitation assay lysis buffer (catalog no. AR0103-100, Beyotime) supplemented with protease inhibitors. A total of 40 μg of the sample was resolved on a 10% SDS–polyacrylamide gel electrophoresis and subsequently transferred onto a polyvinylidene difluoride (PVDF) membrane. Next, the PVDF membrane (catalog no. IPVH00010, Merck Millipore), containing the immobilized protein sample, was immersed in a blocking solution consisting of 5% skim milk (catalog no. 1172GR500, BioFroxx) prepared with Tris-Buffered Saline with Tween (TBST) buffer and sealed for 1 hour. After three washes of the PVDF membrane to remove excess blocker, it was placed into an incubator box containing primary antibodies for overnight incubation. Following three additional washes of the membrane strip to eliminate unbound primary antibodies, secondary antibodies were applied at room temperature for 1 hour. Last, after thoroughly washing away any excess secondary antibody, signals on the membrane activated by enhanced chemiluminescence (ECL) reagents (catalog no. P10300, NCM) were detected using an ECL detection system.

### Single-cell RNA sequencing

For scRNA-seq, heart tissues were obtained from both sham-operated and TAC-treated mice, which were subsequently dissociated into individual cells using a specialized dissociation solution. For each experimental group, single-cell suspensions derived from three to four hearts were combined to create a single sample. The resulting single-cell suspensions were then loaded onto the 10x Chromium platform to facilitate the capture of individual cells using the 10x Genomics Chromium Single-Cell 3′ kit. cDNA amplification, library construction, and sequencing procedures were conducted by LC-BIO Technologies Co. Ltd. (Hangzhou, China).

### Reverse transcription quantitative PCR analysis

TRIzol (catalog no. 15596026CN, Thermo Fisher Scientific) was added to cell and animal samples previously washed with PBS, followed by the extraction of RNA. The extracted RNA was subsequently reverse transcribed into cDNA using a specific reverse transcription kit (catalog no. R333-01, Vazyme). Last, the concentration of cDNA in the samples was quantified using a quantitative PCR (qPCR) instrument (LightCycler 96 SW 1.1), measuring fluorescence signals postamplification, which serves as an indirect indicator of RNA levels in the sample. Primers were purchased from Sangon Biotech (Shanghai, China) as shown in table S9.

### Statistical analysis

Data presented in the current study were representative of a minimum of three independent biological experiments and are expressed as mean ± SD. For in vitro experiments, each dataset represented an average derived from a substantial number of cultured cells. Thus, we assumed that the data followed a normal distribution based on the central limit theorem. In our analysis of in vivo experiments, sample sizes were equal to or greater than six. The normality of each dataset was assessed using the Shapiro-Wilk test. *P* > 0.05 indicated that the data approximated a normal distribution for each group. To identify statistically significant differences between groups, we used two-tailed Student’s *t* tests for comparisons involving two groups and one-way analysis of variance (ANOVA), followed by Tukey’s post hoc test when comparing more than two groups. Data with *P* < 0.05 were deemed to demonstrate statistically significant differences. Statistical analyses were conducted using GraphPad Prism 8.0 software (GraphPad, San Diego, CA).
