# Supplementary material for: Cardiomyocyte-derived YOD1 promotes pathological cardiac hypertrophy by deubiquitinating and stabilizing STAT3
Source: Sci Adv. 2025 Jun 25;11(26):eadu8422. doi: 10.1126/sciadv.adu8422 (PMC12189954; doi:10.1126/sciadv.adu8422)
Supplement: Supplementary file 1 — Figs. S1 to S16 Legends for tables S1 to S9 [file sciadv.adu8422_sm.pdf]

Science Advances
AAAS

Supplementary Materials for

Cardiomyocyte-derived YOD1 promotes pathological cardiac hypertrophy by deubiquitinating and stabilizing STAT3

Bozhi Ye *et al.*

Corresponding author: Guang Liang, wzmcliangguang@163.com; Peiren Shan, prshan@126.com; Gaojun Wu, wugaojun@wzhospital.cn

Sci. Adv. **11**, eadu8422 (2025)
DOI: 10.1126/sciadv.adu8422

The PDF file includes:

Figs. S1 to S16
Legends for tables S1 to S9

Other Supplementary Material for this manuscript includes the following:

Tables S1 to S9

Supplementary Figures:

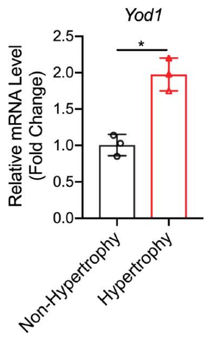

fig. S1: YOD1 mRNA expression in patients with hypertrophy and non-hypertrophy

Real-time qPCR analysis of mRNA expression of *Yod1* in human myocardium tissues from both non-hypertrophic and hypertrophic samples.

**P* < 0.05. n = 3.

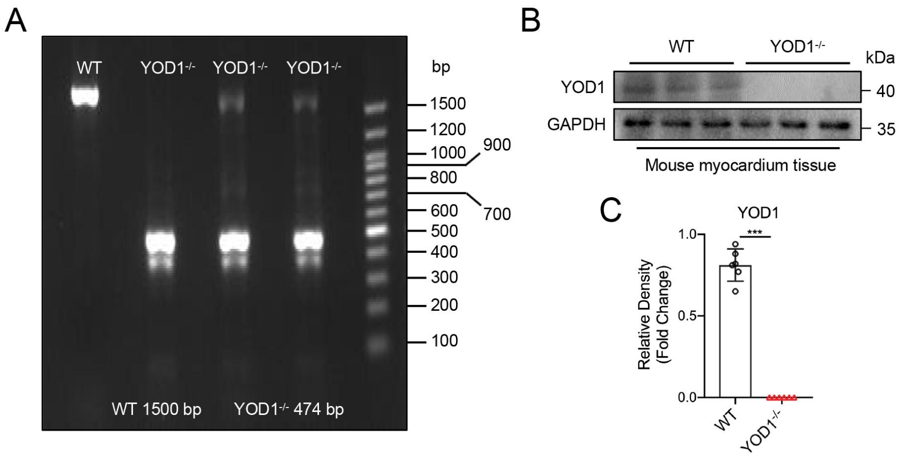

fig. S2: Identification of YOD1 whole-body knockout mice

(**A**) Genetic identification of YOD1 knockout mouse. H_2O served as the blank negative control, while WT refers to the wild-type mouse genotype, and YOD1$^{-/-}$ denotes the YOD1 knockout mouse genotype. The length of the PCR product for the YOD1$^{-/-}$ mouse was found to be 474 bp.

(**B**) Representative western blot analysis of YOD1 in myocardium tissues of YOD1$^{-/-}$ and wild-type mice (B) and densitometric quantification (C).

****P* < 0.001. n = 6.WT: littermate wild-type mice; YOD1$^{-/-}$: whole-body YOD1 knockout mice.

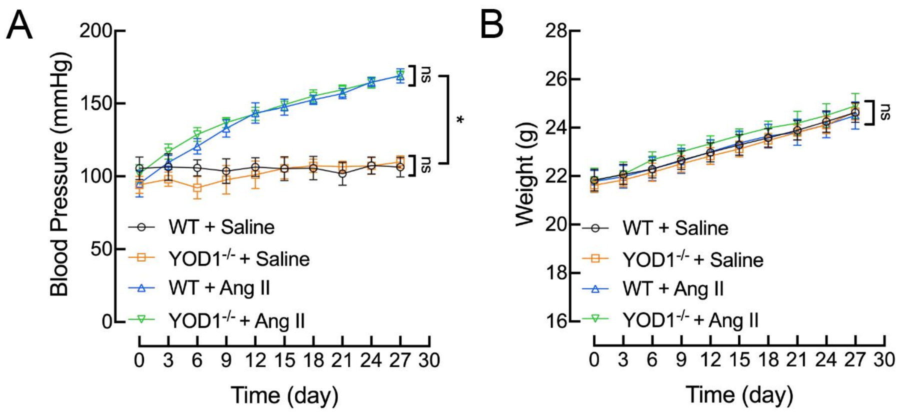

fig. S3: Blood pressure and weight in mice induced by Ang II

(**A-B**) Blood pressure (A) and body weight (B) measuremet of mice in each group.

NS, P > 0.05; *P < 0.05. n = 6. WT: littermate wild-type mice; YOD1$^{-/-}$: whole-body YOD1 knockout mice.

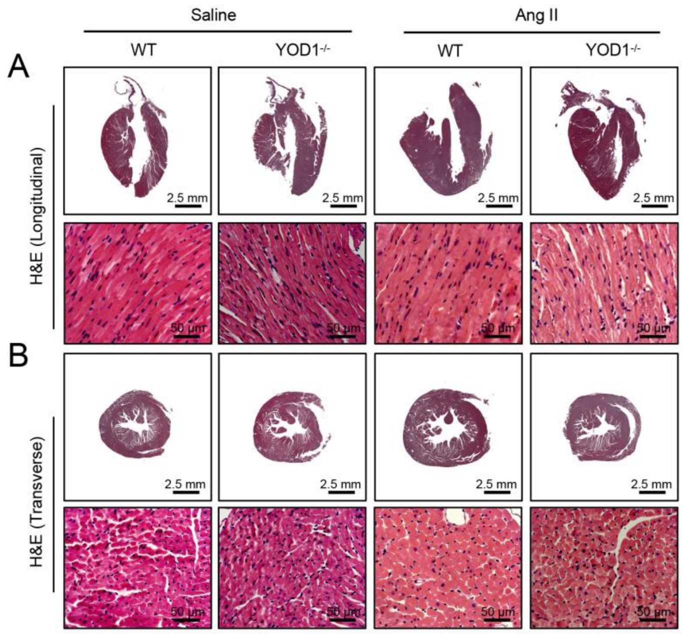

fig. S4: H&E staining of YOD1 knockout mice by Ang II

(**A-B**) Representative images of H&E staining in both longitudinal (A) and transverse (B) sections in myocardium tissues of YOD1$^{-/-}$ and wild-type mice subjected to saline and Ang II stimulation. (scale bar, 2.5 mm and 50 μm)

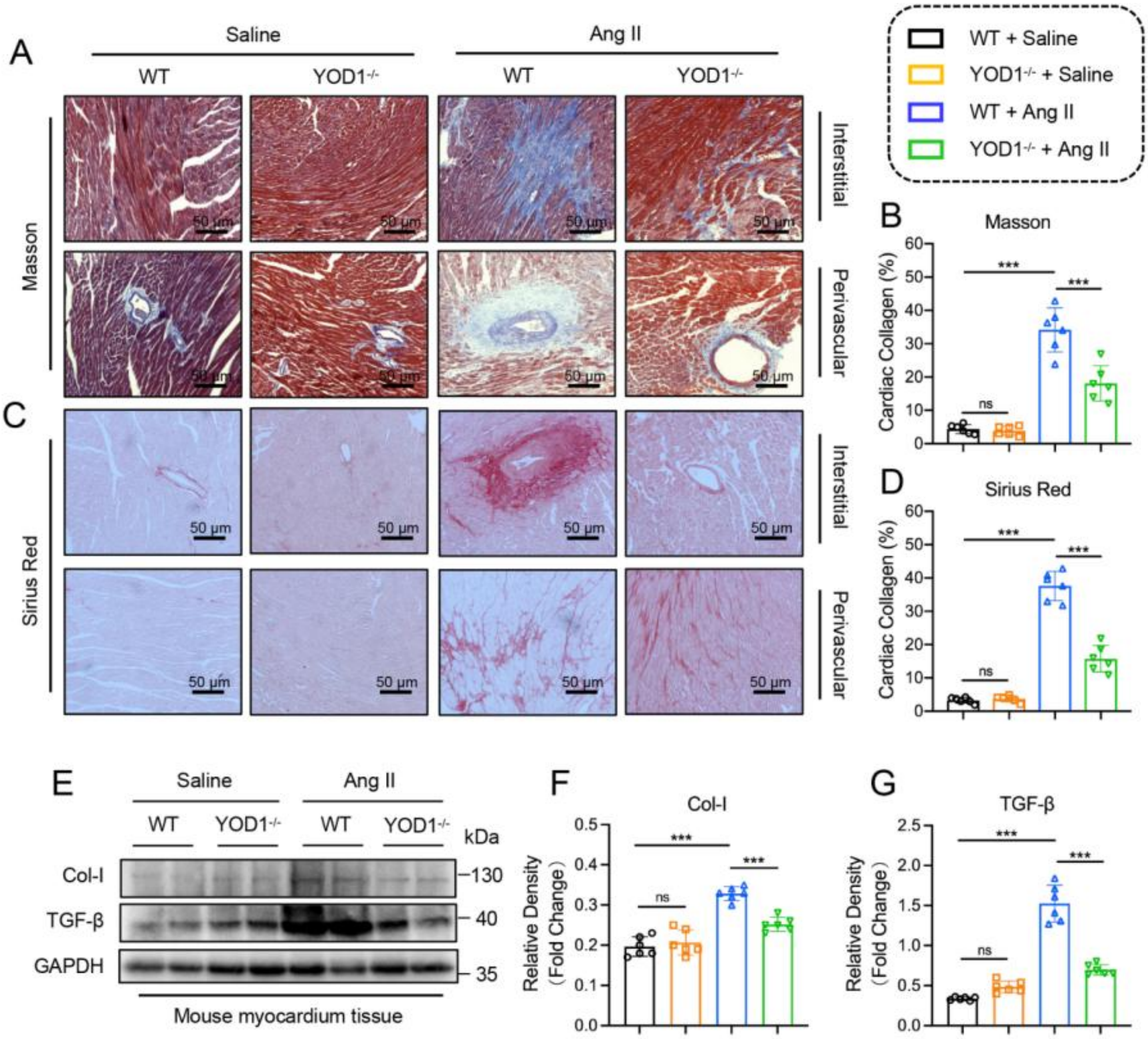

fig. S5: Evaluation of myocardial fibrosis in Ang II-induced YOD1$^{-/-}$ mice

(**A-D**) Fibrotic areas were assessed through Masson's trichrome (A) and Sirius red (C) staining, as well as the quantification of fibrotic regions (B, D) in heart sections from each group. (scale bar, 50 μm).

(**E-G**) Representative western blot analysis of Col-I and TGF-β in myocardium tissues of YOD1$^{-/-}$ and wild-type mice subjected to saline and Ang II stimulation (E) and densitometric quantification (F-G).

NS, P > 0.05; ****P* < 0.001. n = 6. WT: littermate wild-type mice; YOD1$^{-/-}$: whole-body YOD1 knockout mice.

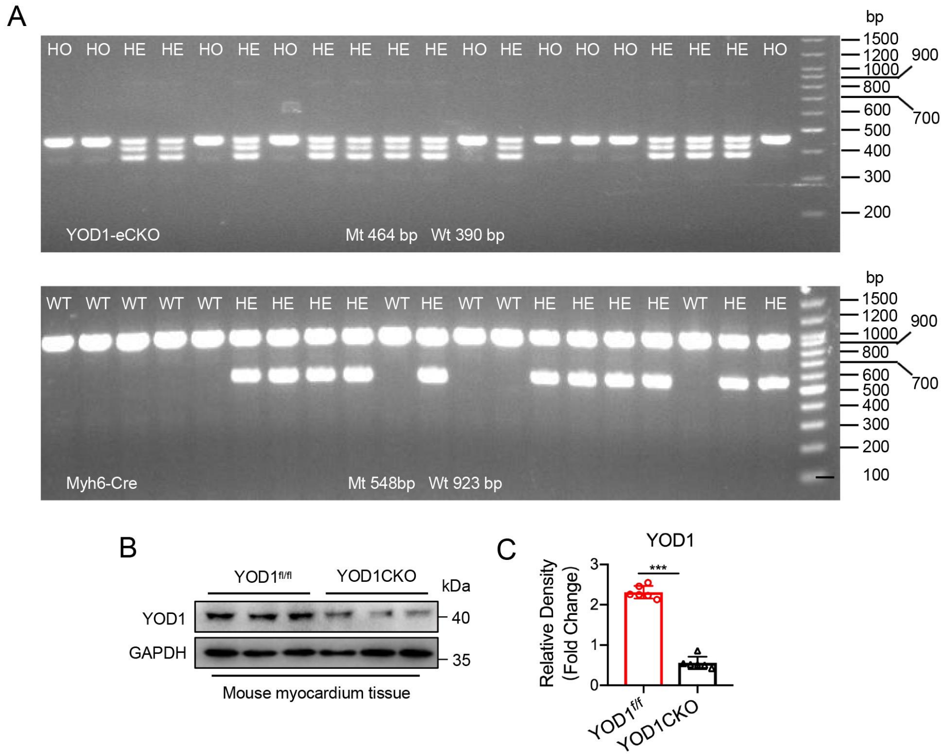

fig. S6: Identification of cardiomyocyte-specific YOD1 knockout mice

(**A**) Genetic identification of YOD1-eCKO mice and Myh6-Cre mice. "HO" denotes homozygous, “HE” indicates heterozygous, while “WT” refers to wild-type mice. The length of the PCR product obtained from YOD1-eCKO mice with a “Wt” genotype measures 390 bp. In contrast, the PCR product length for YOD1-eCKO mice exhibiting an “Mt” genotype is 464 bp. For Myh6-Cre mice, the PCR product length corresponding to a “Wt” genotype is 923 bp, whereas that for an “Mt” genotype is 548 bp.

(**B-C**) Representative western blot analysis of YOD1 in myocardium tissues of $YOD1^{f/f}$ and YOD1CKO mice (B) and densitometric quantification (C).

***P < 0.001. n = 6. $YOD1^{fl/fl}$: $YOD1^{fl/fl}$ mice; YOD1CKO: cardiomyocyte-specific YOD1 knockout mice.

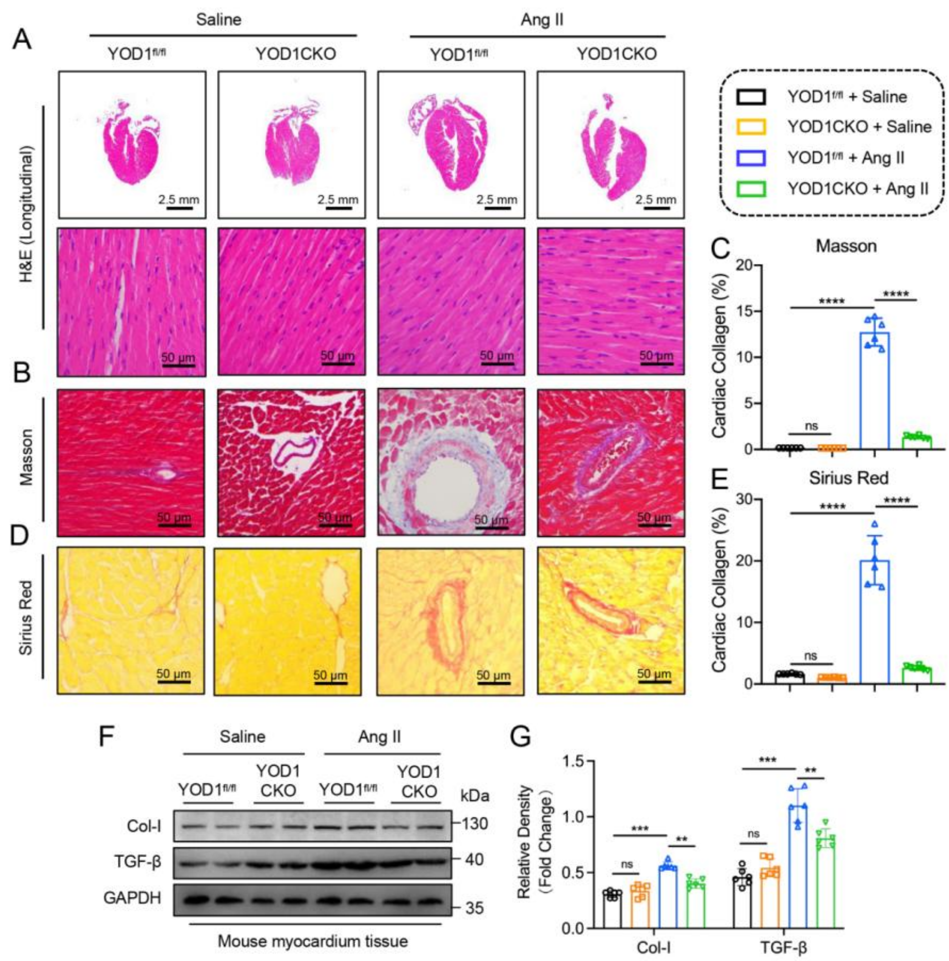

fig. S7： Assessment of ventricular remodeling in cardiomyocyte-specific YOD1 knockout mice

Healthy male YOD1CKO mice (cardiomyocyte-specific YOD1 knockout mice) aged 6-8 weeks and YOD1$^{fl/fl}$ mice were injected with Ang II (1 μg/kg/min) or normal saline via an osmotic pump (cat. no. Alzet MODEL 1004; USA) for 4 weeks to induce cardiac hypertrophy.

(**A**) Representative images of H&E staining in longitudinal sections in myocardium tissues. (scale bar, 2.5 mm and 50 μm)

(**B-E**) Fibrotic areas were assessed through Masson's trichrome (B) and Sirius red (D) staining, as well as the quantification of fibrotic regions (C, E) in heart sections from each group. (scale bar, 50 μm).

(**F-G**) Representative western blot analysis of Col-I and TGF-β in myocardium tissues (F) and densitometric quantification (G).

NS, P > 0.05; *P < 0.05; **P < 0.01; ***P < 0.001. n = 6. YOD1$^{fl/fl}$: YOD1$^{fl/fl}$ mice; YOD1CKO: cardiomyocyte-specific YOD1 knockout mice.

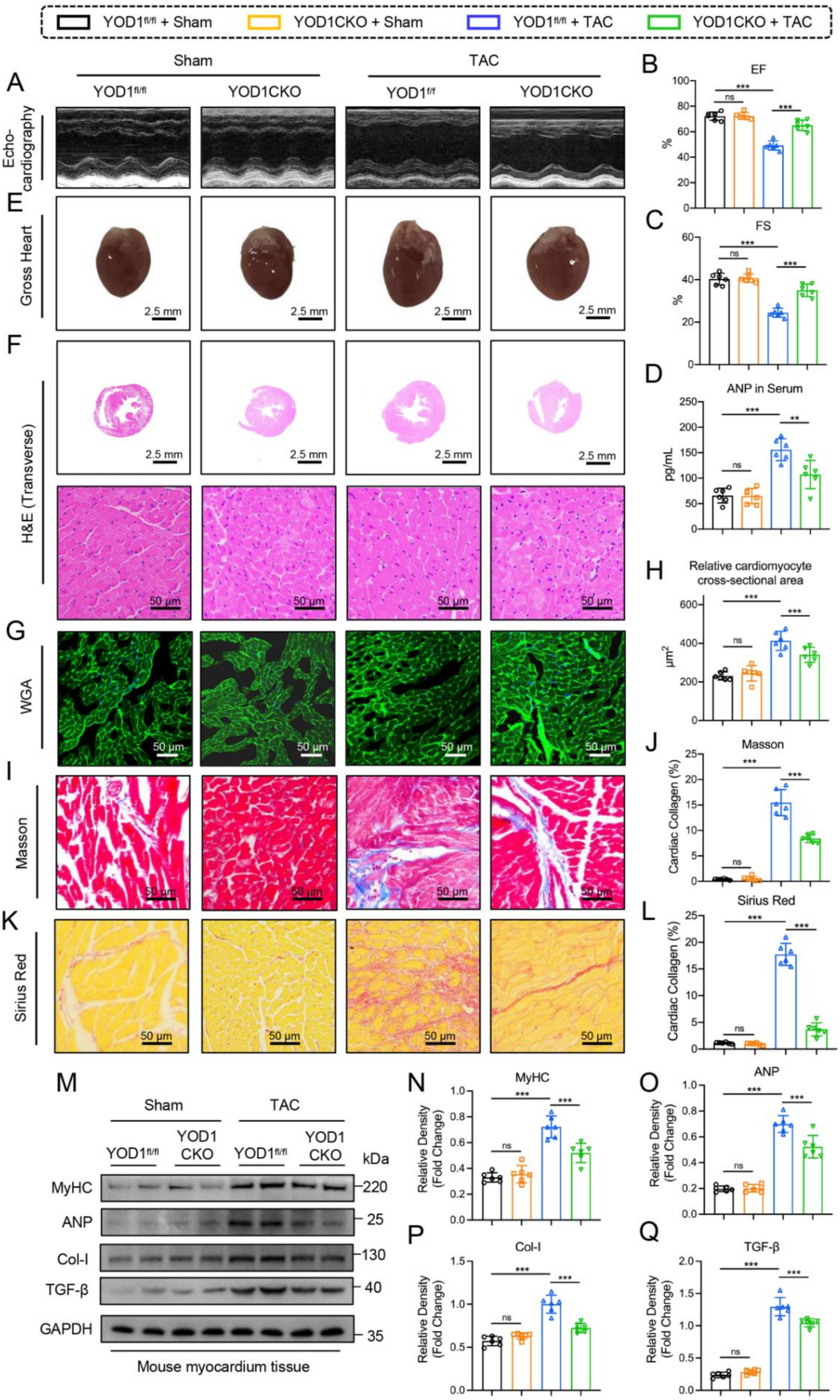

fig. S8: Role of cardiomyocyte YOD1 in TAC-induced ventricular remodeling

Healthy male YOD1CKO mice (cardiomyocyte-specific YOD1 knockout mice) aged 6-8 weeks and YOD1$^{fl/fl}$ mice were subjected to TAC for 6 weeks to induce cardiac hypertrophy.

(**A**) Representative M-mode echocardiography of mice in each group.

(**B-C**) Myocardial function parameters, including ejection fraction (EF, B) and fractional shortening (FS, C), were evaluated in mice through echocardiography.

(**D**) The plasma levels of ANP in each group.

(**E**) Representative images of whole hearts. (scale bar, 2.5 mm).

(**F**) Representative images of H&E staining in transverse section in myocardium tissues. (scale bar, 2.5 mm and 50 μm).

(**G-H**) Representative images of wheat germ agglutinin (WGA, G) staining in sections of hearts and quantitative area analysis (H). (scale bar, 50 μm).

(**I-L**) Fibrotic areas were evaluated using Masson's trichrome (I) and Sirius red (K) staining, as well as the quantification of fibrotic regions (H, L) in heart sections from each group. (scale bar, 50 μm).

(**M-N**) Representative western blot analysis of MyHC, ANP, Col-I and TGF-β (M) and densitometric quantification (N). n = 6.

NS, $P > 0.05$; *$P < 0.05$; **$P < 0.01$; ***$P < 0.001$. n = 6. YOD1$^{fl/fl}$: YOD1$^{fl/fl}$ mice; YOD1CKO: cardiomyocyte-specific YOD1 knockout mice.

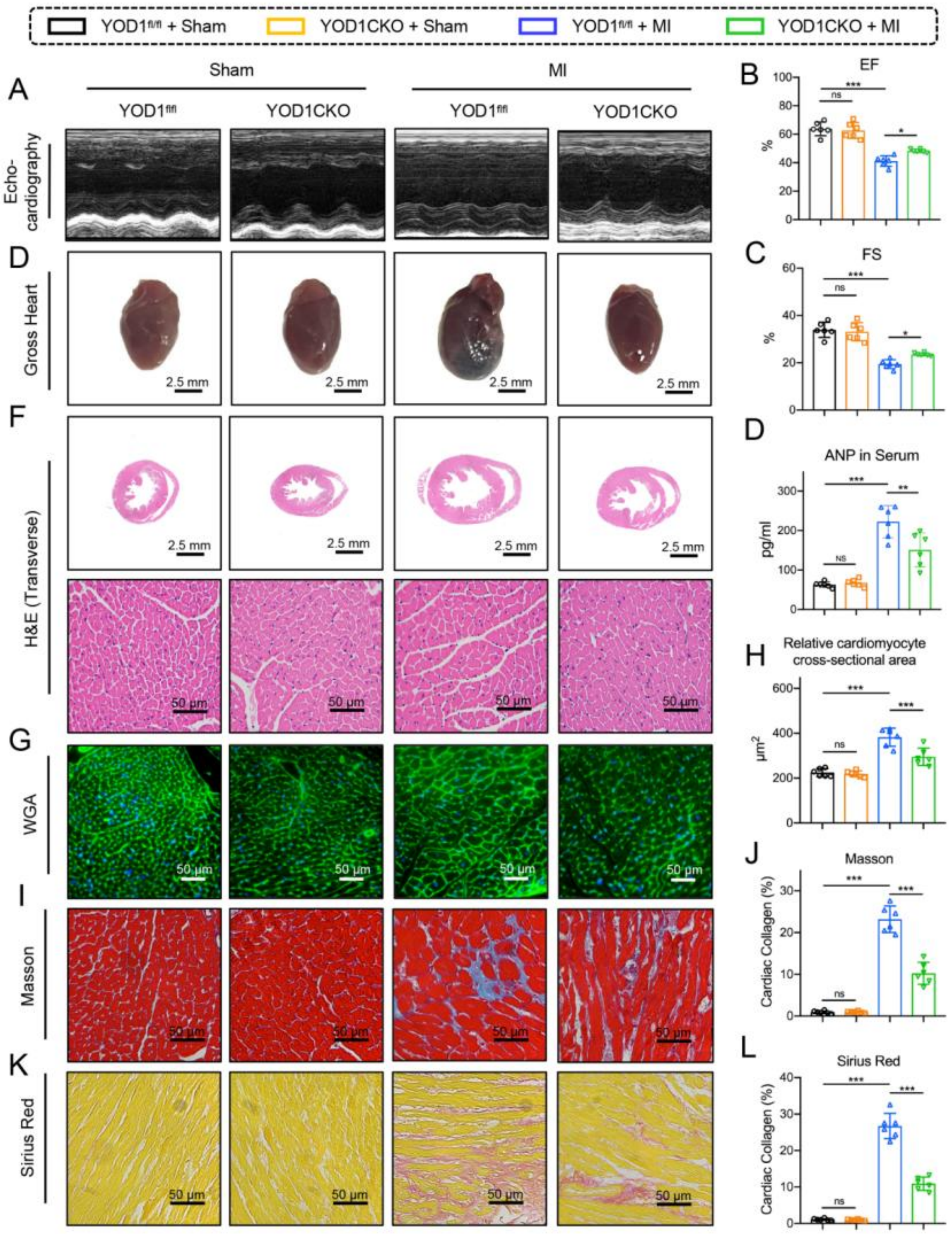

fig. S9: Effects of cardiomyocyte YOD1 in MI-induced ventricular remodeling

Healthy male YOD1CKO mice (cardiomyocyte-specific YOD1 knockout mice) aged 6-8 weeks and YOD1$^{fl/fl}$ mice were subjected to MI for 4 weeks to induce cardiac hypertrophy.

(**A**) Representative M-mode echocardiography of mice in each group.

(**B-C**) Myocardial function parameters, including ejection fraction (EF, B) and fractional shortening (FS, C), were evaluated in mice through echocardiography.

(**D**) The plasma levels of ANP in each group.

(**E**) Representative images of whole hearts. (scale bar, 2.5 mm).
(**F**) Representative images of H&E staining in transverse section in myocardium tissues. (scale bar, 2.5 mm and 50 μm).
(**G-H**) Representative images of wheat germ agglutinin (WGA, G) staining in sections of hearts and quantitative area analysis (H). (scale bar, 50 μm).
(**I-L**) Fibrotic areas were evaluated using Masson's trichrome (I) and Sirius red (K) staining, as well as the quantification of fibrotic regions (H, L) in heart sections from each group. (scale bar, 50 μm).
NS, $P > 0.05$; *$P < 0.05$; **$P < 0.01$; ***$P < 0.001$. n = 6. YOD1$^{fl/fl}$: YOD1$^{fl/fl}$ mice; YOD1CKO: cardiomyocyte-specific YOD1 knockout mice. MI: myocardial infarction.

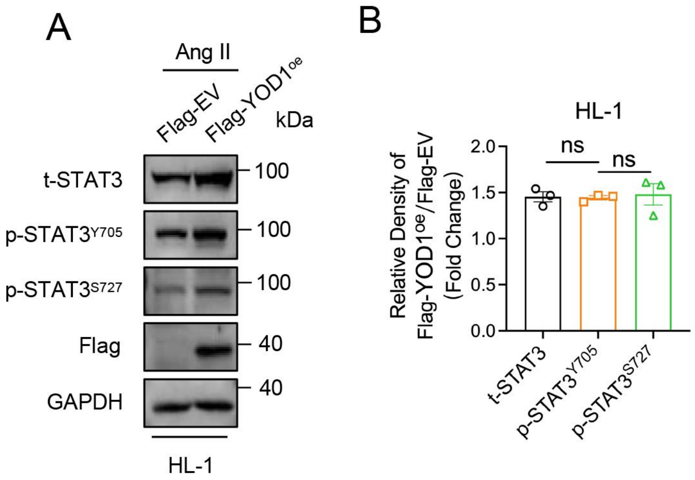

fig. S10: Role of YOD1 on STAT3 phosphorylation in cardiomyocytes

Cardiomyocytes depicted in Supplementary fig. S10A were transfected with plasmids containing either the empty vector (EV) or YOD1 ($YOD1^{oe}$), followed by stimulation with Ang II (1 μM for 24 hours).

(**A-B**) Representative western blot analysis of t-STAT3, $p\text{-}STAT3^{Y705}$ and $p\text{-}STAT3^{Y727}$ in total cell lysate (A) and statistical results (B) .

n = 3. NS, P > 0.05.

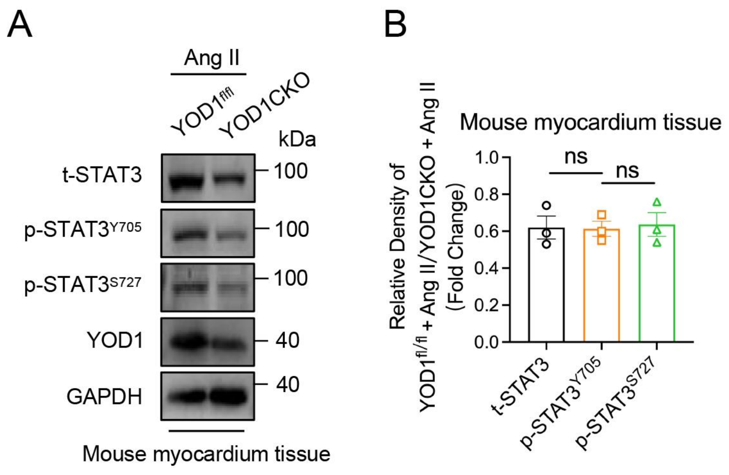

fig. S11：**Effect of YOD1 on STAT3 phosphorylation in myocardium**

Heart tissues depicted in fig. S11A were obtained from healthy male YOD1CKO mice (cardiomyocyte-specific YOD1 knockout mice) aged 6-8 weeks and $YOD1^{fl/fl}$ mice, which were injected with Ang II (1 µg/kg/min) via an osmotic pump (cat. no. Alzet MODEL 1004; USA) for 4 weeks to induce cardiac hypertrophy.

(**A-B**) Representative western blot analysis of t-STAT3, $p\text{-}STAT3^{Y705}$ and $p\text{-}STAT3^{Y727}$ in total heart tissue lysate (A) and statistical results (B) .

n = 3. NS, P > 0.05.

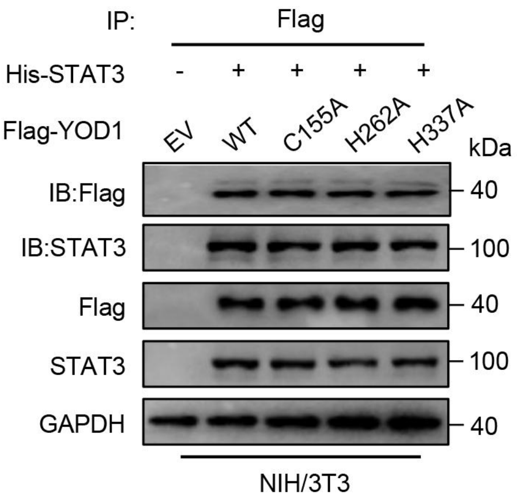

fig. S12: Interaction between site-mutated YOD1 and STAT3

Co-immunoprecipitation of YOD1 was conducted in NIH/3T3 cells that were co-transfected with overexpression plasmids for Flag-YOD1-WT, Flag-YOD1-C155A, Flag-YOD1-H262A, Flag-YOD1-H337A and His-STAT3 plasmids. Exogenous YOD1 was immunoprecipitated by anti-Flag antibody.

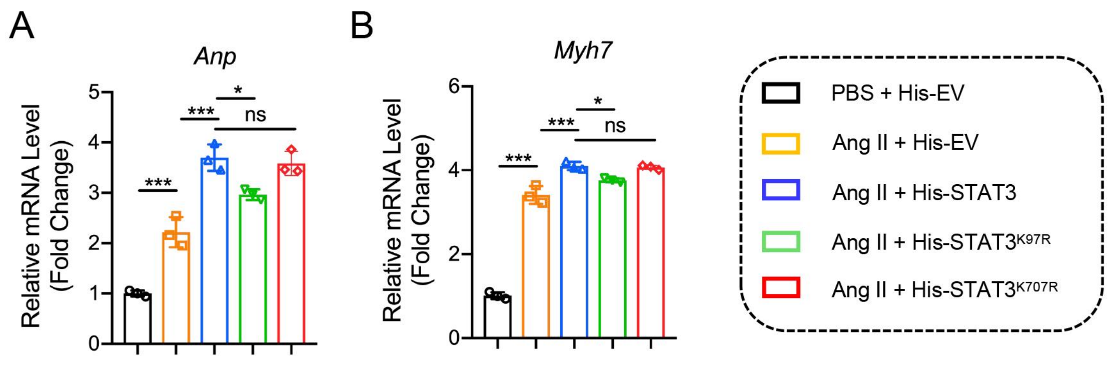

fig. S13: Effect of site-mutant YOD1 on cardiac hypertrophy

Real-time quantitative PCR analysis of mRNA expression levels of *Anp* and *Myh7* in cardiomyocytes induced by Ang II, following transfection with either the wt-STAT3 or mut-STAT3 plasmids. n = 3.

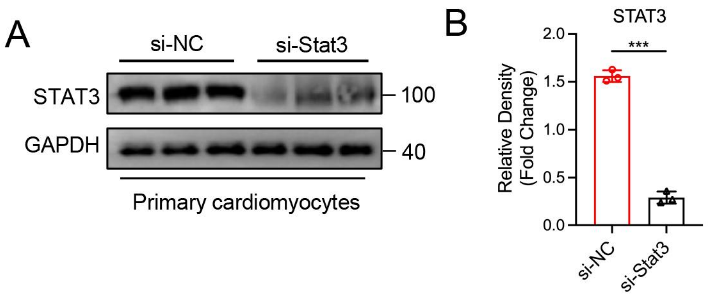

fig. S14: Validation of the siRNA effect on STAT3 interference

(**A-B**) STAT3 knockdown in primary cardiomyocytes was carried out by transfection of siRNA, the expressions of STAT3 protein in primary cardiomyocytes were measured by Western blotting (A) and statistical results (B) (n = 3). ***, $P < 0.001$.

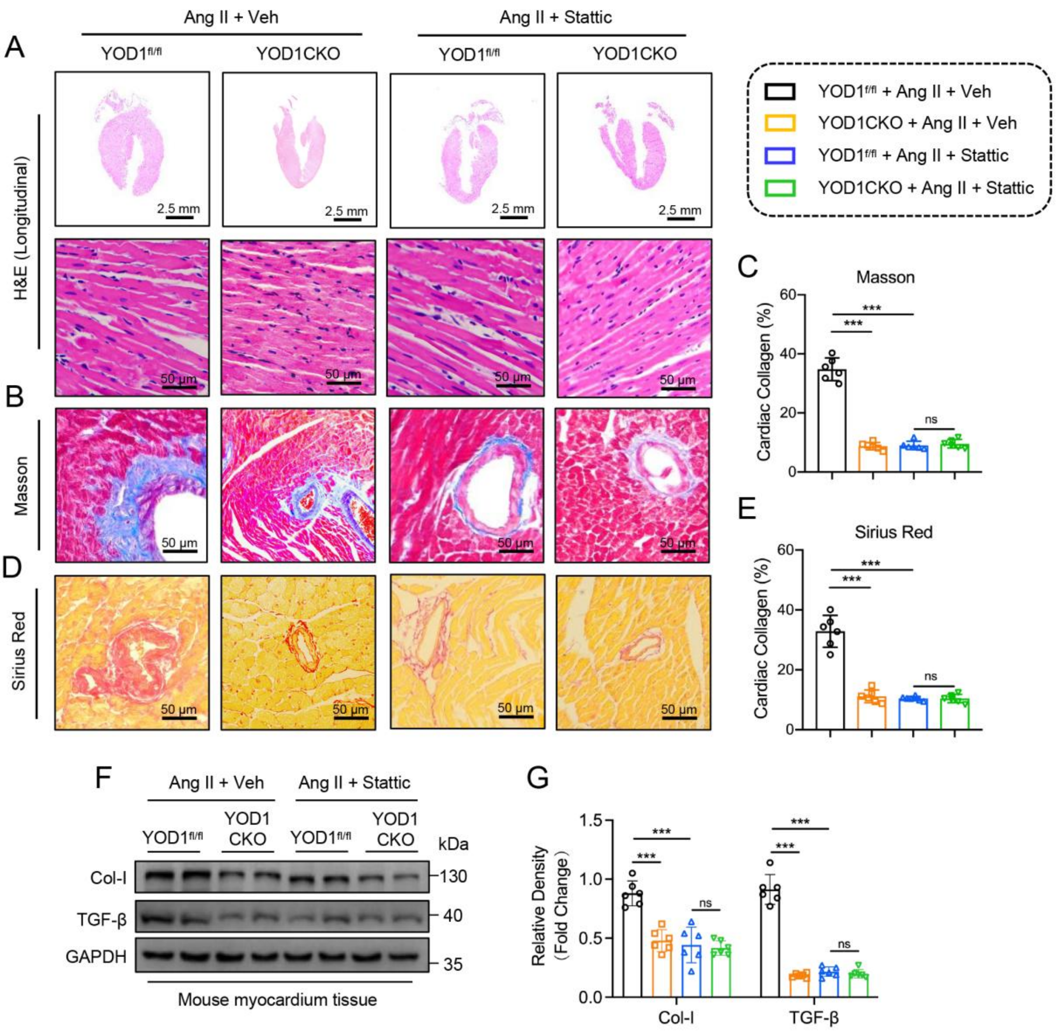

fig. S15: Effects of Stattic on Ventricular Remodeling in YOD1CKO Mice

To verify the effect of YOD1 on STAT3, the STAT3 inhibitor Stattic (10 mg/kg, gavage) or vehicle (0.5% CMC-Na, 0.25% Tween-80) was administered every 3 days via oral gavage. Healthy male YOD1CKO mice (cardiomyocyte-specific YOD1 knockout mice) aged 6-8 weeks and YOD1$^{fl/fl}$ mice were injected with Ang II (1 μg/kg/min) or normal saline via an osmotic pump (cat. no. Alzet MODEL 1004; USA) for 4 weeks to induce cardiac hypertrophy.

(**A**) Representative images of H&E staining in longitudinal sections in myocardium tissues. (scale bar, 2.5 mm and 50 μm)

(**B-E**) Fibrotic areas were assessed through Masson's trichrome (B) and Sirius red (D) staining, as well as the quantification of fibrotic regions (C, E) in heart sections from each group. (scale bar, 50 μm).

(**F-G**) Representative western blot analysis of Col-I and TGF-β in myocardium tissues (F) and densitometric quantification (G).

NS, $P > 0.05$; ***$P < 0.001$. n = 6. YOD1$^{fl/fl}$: YOD1$^{fl/fl}$ mice; YOD1CKO: cardiomyocyte-specific YOD1 knockout mice.

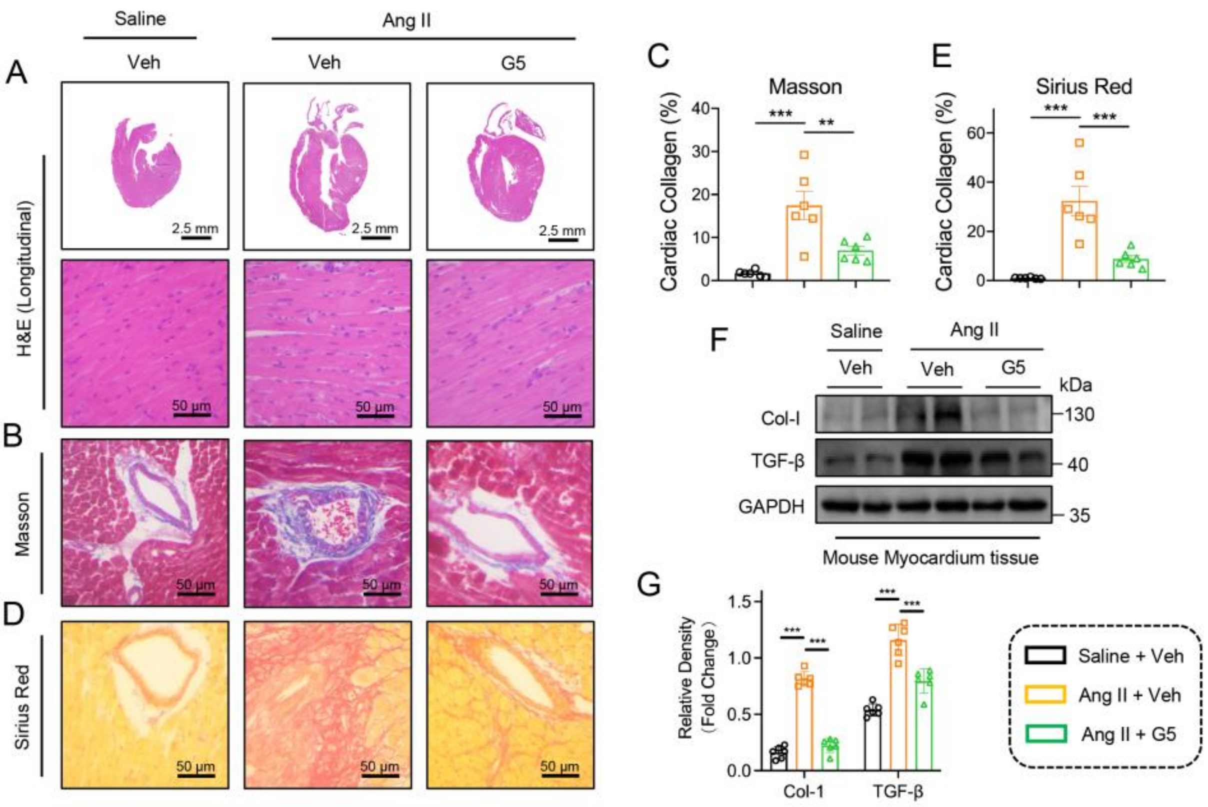

fig. S16: Effects of G5 on Ventricular Remodeling in Mice

To invvestigate the pharmacological inhibition of YOD1 on cardiac hypertrophy, the YOD1 inhibitor G5 (10 mg/kg, gavage) or vehicle (0.5% CMC-Na, 0.25% Tween-80) was delivered every 3 days using oral gavage. Healthy male wild type mice aged 6-8 weeks were injected with Ang II (1 μg/kg/min) or normal saline via an osmotic pump (cat. no. Alzet MODEL 1004; USA) for 4 weeks to induce cardiac hypertrophy.

(**A**) Representative images of H&E staining in longitudinal sections in myocardium tissues. (scale bar, 2.5 mm and 50 μm)

(**B-E**) Fibrotic areas were assessed through Masson's trichrome (B) and Sirius red (D) staining, as well as the quantification of fibrotic regions (C, E) in heart sections from each group. (scale bar, 50 μm).

(**F-G**) Representative western blot analysis of Col-I and TGF-β in myocardium tissues (F) and densitometric quantification (G).

NS, $P > 0.05$; $^{**}P < 0.01$; $^{***}P < 0.001$. n = 6.

Table S1. (separate file)
Spreadsheet containing biometric and echocardiographic parameters in Ang II-challenged mouse experiment. Osmotic pumps filled with normal saline or Ang II (1 μg/kg/min) were implanted in $YOD1^{-/-}$ mice, along with their respective control litters, for a duration of four weeks.

Table S2. (separate file)
Spreadsheet containing biometric and echocardiographic parameters in Ang II-challenged mouse experiment. Osmotic pumps filled with normal saline or Ang II (1 μg/kg/min) were implanted in Cardiomyocyte-specific knockout of YOD1 mice (YOD1CKO), along with their respective control litters ($YOD1^{fl/fl}$), for a duration of four weeks.

Table S3. (separate file)
Spreadsheet containing biometric and echocardiographic parameters in TAC-challenged mouse experiment. Healthy YOD1CKO and $YOD1^{fl/fl}$ male mice, aged 6 to 8 weeks, were selectively chosen for either TAC or sham surgery, with a duration of four weeks.

Table S4. (separate file)
Spreadsheet containing biometric and echocardiographic parameters in MI-challenged mouse experiment. Healthy YOD1CKO and $YOD1^{fl/fl}$ male mice, aged 6 to 8 weeks, were selectively chosen for either MI or sham surgery, with a duration of four weeks.

Table S5. (separate file)
Spreadsheet containing data from interactome, ubiquitinome, and proteome analyses conducted on cardiomyocytes that were transfected with either the YOD1 plasmid or a control plasmid.

Table S6. (separate file)
Spreadsheet containing biometric and echocardiographic parameters in Ang II-challenged mouse experiment. The STAT3 inhibitor Stattic (10 mg/kg) were administered orally every two days in YOD1CKO and $YOD1^{fl/fl}$ mice, followed by Ang II (1 μg/kg/min) treatment, which was infused via osmotic pump for a duration of four weeks.

Table S7. (separate file)
Spreadsheet containing biometric and echocardiographic parameters in Ang II-challenged mouse experiment. The YOD1 inhibitor G5 (10 mg/kg) were administered orally every two days in wild type mice, followed by Ang II (1 μg/kg/min) treatment, which was infused via osmotic pump for a duration of four weeks.

Table S8. (separate file)
Spreadsheet containing the clinical characteristics of the patients with cardiac hypertrophy and non-cardiac hypertrophy (related to Fig. 1D).

Table S9. (separate file)
Spreadsheet containing primer sequences used for real-time qPCR analysis.
